# WMRE2030: integrating wearable devices, multi-omics, and artificial intelligence–driven real-time feedback into a daily-scale closed-loop framework for a new era of precision exercise

**DOI:** 10.3389/fphys.2026.1883295

**Published:** 2026-09-21

**Authors:** Mingrui Wang, Lun Chen, Mingyue Yin, Bing Zhang, Jilong Wu, Haowei Sun, Linghua Yan, Bing Wei, Feng Zhang, Cunjian Bi, Jun Wang, Shaoliang Zhang

**Affiliations:** 1 Department of Exercise Physiology, Beijing Sport University, Beijing, China; 2 Division of Sports Science and Physical Education, Tsinghua University, Beijing, China; 3 Top -Tier Performance Physical Fitness Training Center, Beijing, China; 4 The Mary MacKillop Institute for Health Research, Australian Catholic University, Melbourne, VIC, Australia; 5 Xiaomi Communications Technology Co., Ltd., Beijing, China; 6 Beijing Deepwise Technology Co., Ltd., Beijing, China; 7 Department of Innovative Technology, Shanghai Tongshu Biotechnology Research Institute, Shanghai, China; 8 Competitor Institute of Sports and Nutrition (CISN), Beijing, China; 9 School of Sports Science and Engineering, East China University of Science and Technology, Shanghai, China; 10 School of Physical Education, Chizhou University, Chizhou, China; 11 Athletic Performance and Data Science Laboratory (APDS), Division of Sports Science and Physical Education, Tsinghua University, Beijing, China

**Keywords:** artificial intelligence, closed-loop feedback, digital health, exercise physiology, multi-omics, personalised intervention, precision exercise, wearable devices

## Abstract

Precision exercise is increasingly supported by wearable technologies, multi-omics profiling, and artificial intelligence; however, these components are often applied in isolation, limiting their capacity to support continuous and interpretable decision-making in real-world settings. This review proposes WMRE2030, a day-scale closed-loop methodological framework integrating Wearables (W), Multi-omics (M), AI-driven Real-Time Feedback (R), and Exercise (E). Within this architecture, wearable devices continuously capture physiological, behavioural, and contextual states; multi-omics provides relatively stable or periodically updated biological background, response potential, and safety constraints; artificial intelligence organizes heterogeneous information into evidence-constrained and traceable decision support; and exercise functions as the executable intervention whose outcomes are returned to the system for iterative updating. The framework emphasizes “the same architecture, different parameters, “ allowing sensors, omics inputs, decision thresholds, and levels of professional oversight to be adapted across clinical populations, the general population, and high-performance athletes. WMRE2030 should currently be regarded as a testable methodological roadmap rather than a validated autonomous prescription system. Future research should evaluate its incremental value through longitudinal, micro-randomized, and multicentre studies, while addressing interoperability, privacy, algorithmic transparency, safety brakes, cost-effectiveness, scalability, and equitable access. By connecting biological interpretation with continuous sensing and adaptive decision support, WMRE2030 may provide a practical pathway toward more reliable and sustainable precision exercise.

## 1 Introduction

With advances in information technology and life sciences, key enabling technologies—including wearable devices, artificial intelligence, and multi-omics profiling—have progressively matured, allowing continuous characterization of inter-individual variability and providing a practical foundation for the implementation of precision exercise, an individualized and adaptive approach that tailors exercise prescription and its subsequent adjustment to individual characteristics, risk profiles, and observed responses (Ross et al., 2019). From the broader perspective of digital health, related solutions have also expanded rapidly worldwide, progressing from simple data recording toward broader applications in health management and behavioural intervention and thereby offering new opportunities to improve the efficiency, accessibility, and equity of health services (Kane, 2024; Singh et al., 2024; Organization, 2025). However, their effectiveness remains highly dependent on integration with healthcare system planning and governance frameworks (Jandoo, 2020; WHO, 2025).

At the technological level, wearable devices have been widely adopted in daily health management, enabling continuous, low-burden monitoring of physiological and behavioural characteristics (Li et al., 2017; Dunn et al., 2021; Patten et al., 2026). This has provided a novel data foundation for dynamically capturing training load, recovery processes, and lifestyle-related patterns (Canali et al., 2022). Meanwhile, artificial intelligence technologies are increasingly being incorporated into health interventions and clinical research, reshaping both study design and practical implementation. The introduction of reporting guidelines such as SPIRIT-AI and CONSORT-AI highlights the transition of AI from an auxiliary analytical tool to a core component of intervention systems, with its application now subject to explicit methodological standards (Liu et al., 2020; Rivera et al., 2020). In parallel, multi-omics approaches are widely used in precision health research to characterize individual variability, adaptive potential, and risk profiles, serving as a key tool for understanding biological and behavioural heterogeneity (Babu and Snyder, 2023). Notably, the declining cost and expanding accessibility of multi-omics technologies, along with the rise of direct-to-consumer testing, have fundamentally transformed how individuals access personal biological information (Knoppers et al., 2021; NHGRI, 2023).

Despite these advances in data acquisition and intelligent analytics, the exercise and health sciences still lack a systematic methodological framework for integrating multi-source technologies and individual-level information to support stable decision-making in real-world settings. In contrast, the fields of medicine and digital health have gradually established frameworks centred on “multi-source data integration—personalized decision-making—continuous management.” Within this context, digital twins have evolved from conceptual constructs toward practical applications, enabling the integration of multidimensional individual data and supporting prospective decision-making (Vallée, 2024). Related studies have also emphasized the synergistic integration of multi-omics, wearable monitoring, and artificial intelligence to support prevention and chronic disease management (Guasch-Ferré et al., 2025; Ooka, 2025). By comparison, although exercise science and sports training have explored closed-loop training systems and machine learning–based risk identification (Nasb et al., 2025; Tian et al., 2025), the current evidence base remains fragmented, with substantial heterogeneity in study populations, outcome definitions, predictor variables, and modelling procedures, together with scarce external validation and limited model transparency and interpretability (Van Eetvelde et al., 2021; Bullock et al., 2022; Leckey et al., 2025). Moreover, the concept of a “sports digital twin” largely remains at a theoretical stage (Hliš et al., 2024). Overall, existing frameworks in the exercise domain are often task-specific or locally closed-loop, and are insufficient to support multi-source integration and sustained decision-making, highlighting a critical methodological gap.

Against this background of evolving health policy, technological advancement, and methodological challenges, a key question emerges: how to integrate wearable devices, artificial intelligence, and multi-omics information into a decision-support system that can operate reliably at the day scale across diverse populations. To address this, the present study proposes the WMRE2030 methodological framework for precision exercise (Wearables; Multi-omics; AI-driven Real-Time Feedback; Exercise), aiming to provide a structured pathway that is reproducible, scalable, and constrained by evidence-based principles and safety boundaries.

## 2 WMRE2030: from technological assembly to an operational system

### 2.1 Framework definition and core modules

Building on these technological advances and methodological challenges, the key issue in precision exercise is progressively shifting from the application of individual technologies toward the systematic integration of heterogeneous data sources and technological components to establish stable, maintainable, and scalable closed-loop decision-support systems. Experiences in the digital health field indicate that when continuous data streams are integrated into intervention and management systems, the key to ensuring long-term stable operation is not the scale of the data but how data acquisition, interpretation, decision-making, and feedback are structurally connected. The organization of data-decision flows directly impacts the sustainability of the system (Pannunzio et al., 2023).

In this context, the WMRE2030 precision exercise methodological integration framework proposed herein is not simply a repackaging of a single technology but a systematic approach designed for real-world implementation. Its core lies in integrating continuous sensing, biological explanations, computational decision-making, and prescription execution into a sustainably updating closed-loop structure, with modular design to accommodate cross-population parameterized adaptations. This approach is in line with the overarching concept of digital twins in the health field, where multi-source data streams and mechanism or statistical models are integrated to create dynamic systems that update over time and support intervention decisions (Katsoulakis et al., 2024). Within this framework, W (Wearables) serves as the continuous sensing layer, providing physiological and behavioral data streams from wearable devices. Its function is to act as an “input layer” rather than a “conclusion layer, “ requiring systematic translation and quality control to transform “accessible” data into “usable” digital biomarkers (Daniore et al., 2024); M (Multi-omics) acts as the biological explanation and constraint layer, capturing the individual’s capability background, response potential, and risk boundaries in a low-frequency manner, providing the decision-making process with biological applicability and extrapolation limits (Liu et al., 2025a); R (AI-driven Real-time Feedback) takes on the computational and decision-organizing role, integrating W’s continuous states, M’s low-frequency constraints, and situational information into structured, traceable decision support that is bound by safety limits and not replacing the prescription itself. This positioning is also consistent with the systemic organizational experiences of digital twins and intelligent training in sports (Hliš et al., 2024); E (Exercise) corresponds to the output, execution, and feedback of exercise prescriptions. Its results feed back into the system through W and are adjusted through M’s periodic calibration when necessary, completing the closed-loop update. Through the division of labor among these modules and the organization of the information flow, WMRE2030 aims to provide a functional, scalable, and auditable methodological framework for precision exercise.

### 2.2 WMRE2030 operational process: a real-world daily-scale closed-loop mechanism

WMRE2030 operates with a daily-scale unit at its core, continuously integrating multi-source physiological, behavioral, and situational information to support the dynamic generation, execution, and calibration of exercise interventions in real-world contexts, providing the operational methodological foundation for implementing precision exercise prescriptions. This framework incorporates individual multi-omics data (background and dynamic layers), wearable device (real-time layer) physiological and behavioral signals, and daily situational factors into a unified decision chain, creating a closed-loop structure that updates with individual status changes (**Figure 1**).

**Figure 1:**
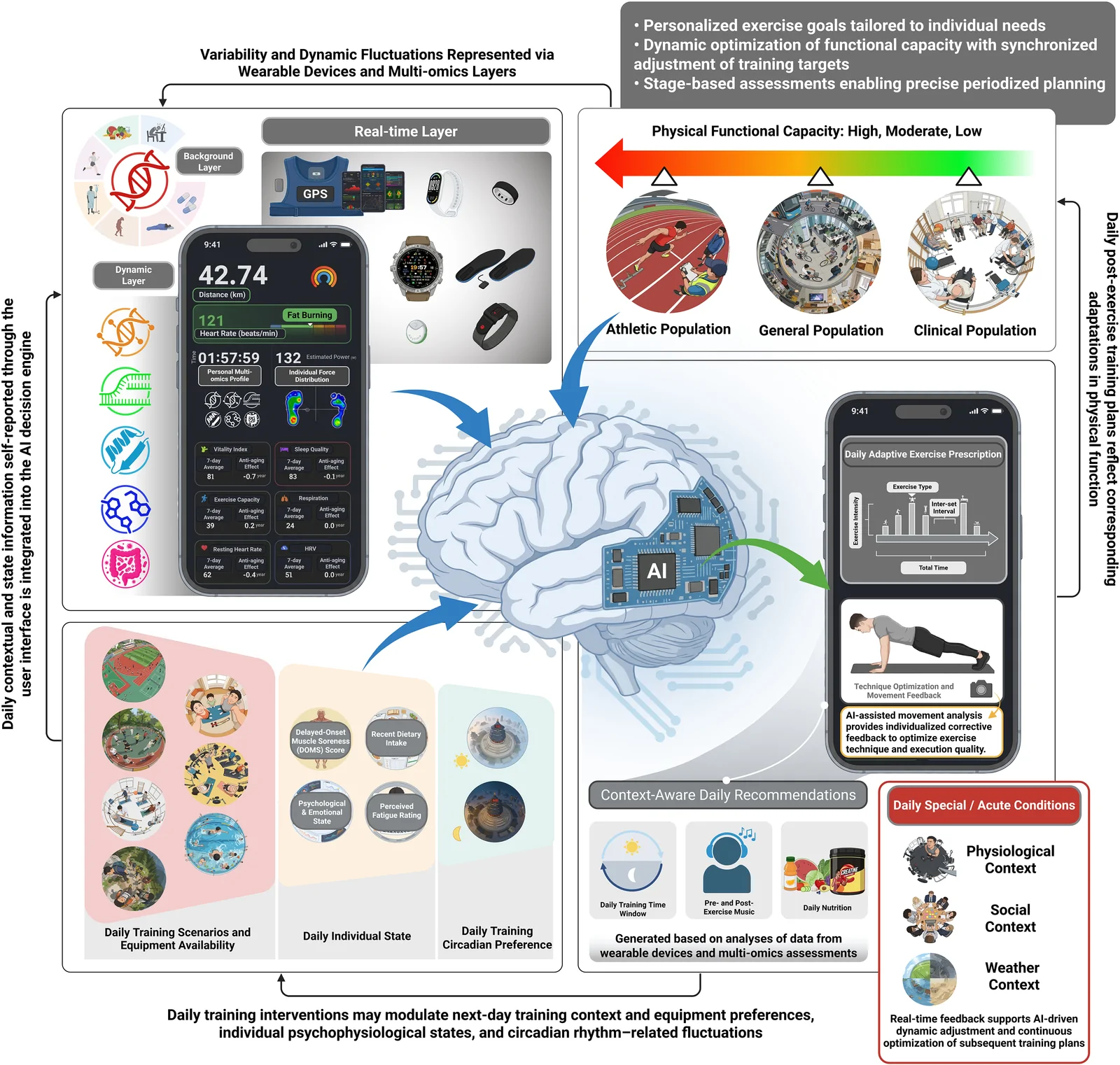
Day-scale closed-loop operating mechanism of the WMRE2030 precision exercise methodological framework.

In the multi-omics data, genetic background is a relatively stable feature that only needs to be obtained once, while other omics layers are incorporated into the model in a low-frequency, periodic manner to describe an individual’s capability base, adaptive potential, and potential risk constraints. Correspondingly, the continuous data collected by wearable devices forms the dynamic input of the system, reflecting the individual’s real-time state and its fluctuation trajectory in both daily life and training environments. At the same time, daily situational information, including training settings, equipment conditions, subjective feelings, circadian rhythm features, and environmental factors, is synchronized and integrated to reduce the likelihood of short-term fluctuations being misinterpreted as adaptive changes or risk signals.

Subsequently, the aforementioned multi-layered information is incorporated into structured analysis within the computational and decision organization module. This module adapts intervention goals and risk tolerance based on the individual’s fitness level and population attributes. Within the biological background constraints, the system performs a comprehensive evaluation of the daily status and dynamically weighs training benefits, recovery needs, and safety boundaries to determine whether to continue with the current training direction or make adjustments to training intensity, volume, and form. When necessary, more conservative intervention strategies are introduced. This decision-making process, guided by multi-objective coordination, ensures that the system continuously updates the intervention logic as individual states evolve.

The exercise prescriptions generated by this process are defined as executable action units within the closed loop, with their significance lying in the fact that their execution results can be continuously sensed and fed back into the system. After the daily intervention is completed, relevant physiological and behavioral responses are monitored through wearable devices, entering the next decision cycle. The system also flags any special or unexpected situations that may arise during the day and triggers immediate adjustments when necessary. Thus, a single intervention not only impacts the current day’s status but also influences future training scenario selection, load distribution, and recovery strategies through the state feedback mechanism, facilitating the continuous evolution of status, decision-making, and execution across time.

### 2.3 Real-world experimental extensions, application scenarios, and a prudent implementation pathway

As a methodological framework, WMRE2030 does not correspond to a single fixed experimental paradigm; rather, it provides a combinable structural space for diverse, multi-level study designs and real-world applications. In future empirical research, this framework can support multiple comparative and extension pathways. On the one hand, traditional standardized prescriptions (“one-size-fits-all” training) can be compared in parallel with precision exercise strategies based on daily-scale closed-loop systems, allowing systematic evaluation of the incremental value of continuous decision-making in terms of training effectiveness, stability, and safety. On the other hand, within the domain of precision exercise itself, comparative studies can be conducted across different AI implementation pathways (e.g., rule-dominant, model-driven, or hybrid strategies) to clarify their respective applicability boundaries across populations and scenarios.

At the technical level, WMRE2030 also provides flexibility for comparative configurations of multi-source information. For example, studies may explore optimal trade-offs between cost, accessibility, and decision gains by incorporating different types of wearable devices, combinations of physiological signals, or progressively adding layers of omics data. Such investigations are not limited to elite athletes but can be extended to general populations and specific health groups, thereby testing the feasibility and robustness of the “same framework, different parameters” principle in cross-population applications. As a result, future experimental designs are unlikely to converge toward a single pathway but will instead evolve along multidimensional and progressive trajectories.

It is important to emphasize that, precisely because WMRE2030 is intended for long-term operation in real-world contexts, its implementation must be embedded within clear safety and ethical boundaries. At the current methodological stage, with relevant technologies still rapidly evolving, any automated or semi-automated decision outputs should not operate without continuous oversight by researchers, coaches, or healthcare professionals. This is particularly critical in scenarios involving high-risk populations, complex health conditions, or novel model configurations. Systems should incorporate explicit “safety brake” mechanisms to prevent potential risks arising from transient fluctuations, algorithmic bias, or inappropriate extrapolation. This cautious stance does not negate the potential of the technology but ensures that evidence is accumulated progressively under controlled conditions.

Based on this foundation, it can be anticipated that, with the maturation of wearable technologies, decreasing costs of multi-omics, and the standardization of AI methodologies, the daily-scale closed-loop paradigm represented by WMRE2030 will provide broader opportunities for exploration in precision exercise research and practice. Through stepwise experimental validation and scenario expansion under appropriate regulatory and methodological constraints, this framework has the potential to serve as a critical bridge between methodological innovation and real-world implementation, thereby establishing a solid foundation for the long-term development of precision exercise.

## 3 Trends in core technological domains underpinning WMRE2030

To systematically characterize the technological foundations underpinning the WMRE2030 framework, we performed a descriptive publication-trend retrieval and visualization analysis across four core domains—artificial intelligence, multi-omics, wearable technologies, and precision exercise—over the period 2010–2025 using databases with complementary disciplinary coverage (**Figure 2**). Each database was searched independently using predefined domain-specific Boolean queries combining the principal terms for each domain with commonly used variants and related terminology. Records were included when they were indexed in the corresponding database between 2010 and 2025 and matched the predefined search query for the relevant domain. The complete database-specific search strings, search fields, and applicable restrictions are presented in **Table 1**, thereby allowing the retrieval process to be transparently evaluated and reproduced.

**Figure 2:**
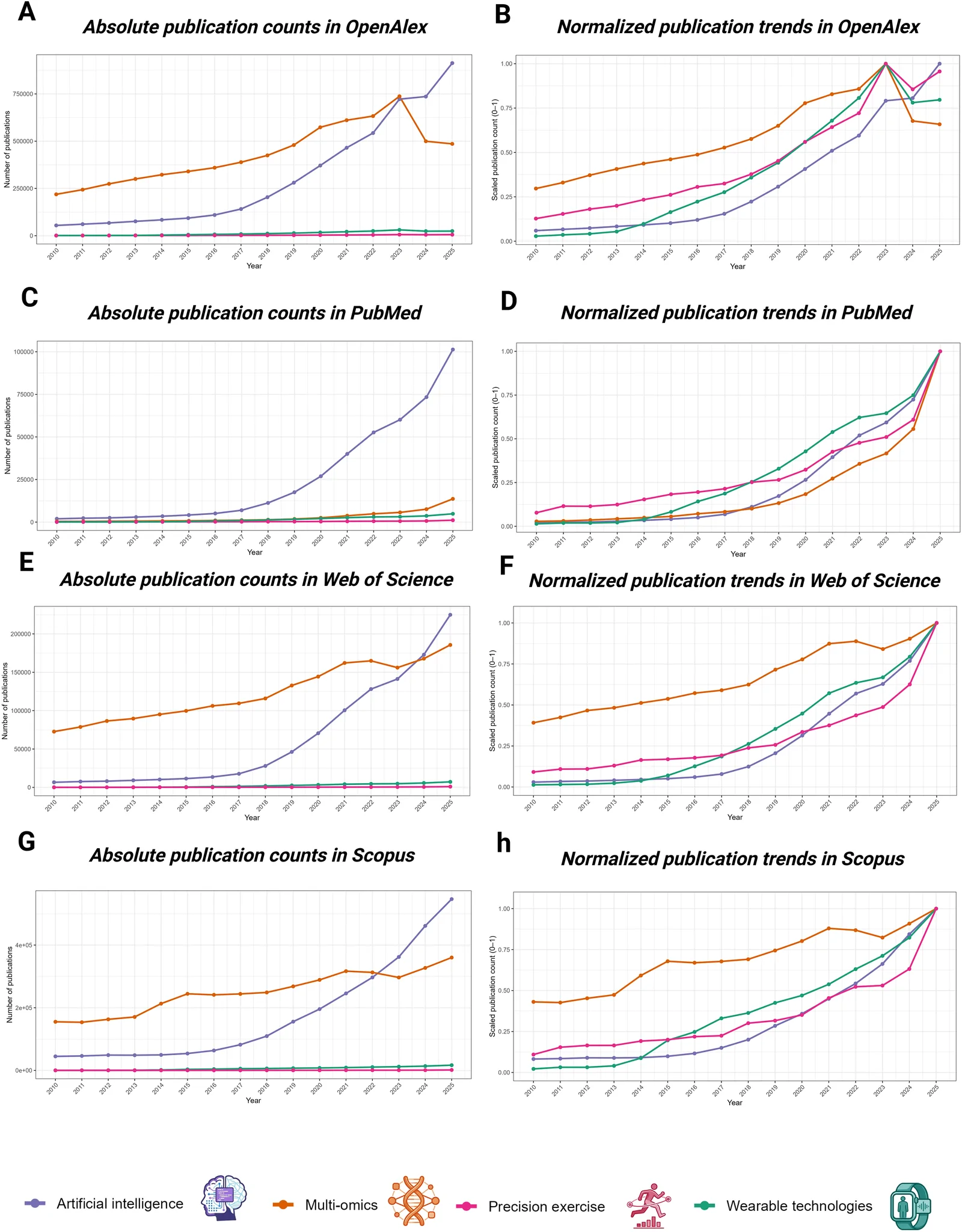
Publication trends of core WMRE2030-related domains across four major databases, 2010 -2025. **(A)** Absolute publication counts in OpenAlex; **(B)** normalized publication trends in OpenAlex; **(C)** absolute publication counts in PubMed; **(D)** normalized publication trends in PubMed; **(E)** absolute publication counts in Web of Science; **(F)** normalized publication trends in Web of Science; **(G)** absolute publication counts in Scopus; and **(H)** normalized publication trends in Scopus.

**Table 1:** Full search strategies for trend analysis.

Domain	Conceptual scope	Database	Search query
Artificial Intelligence (including digital twins)	Artificial intelligence-based modelling, prediction, and decision-support systems in health, sport, and exercise science. Digital twin-related concepts were included within this domain.	OpenAlex	(“artificial intelligence” OR “machine learning” OR “deep learning” OR “neural network” OR “large language model” OR “generative ai” OR “reinforcement learning” OR “computer vision” OR “decision support system” OR “digital twin” OR “digital twins” OR “digital twinning”)
		Web of Science	TS=(“artificial intelligence” OR “machine learning” OR “deep learning” OR “neural network” OR “large language model” OR “generative ai” OR “reinforcement learning” OR “computer vision” OR “decision support system” OR “digital twin” OR “digital twins” OR “digital twinning”)
		Scopus	TITLE-ABS-KEY(“artificial intelligence” OR “machine learning” OR “deep learning” OR “neural network” OR “large language model” OR “generative ai” OR “reinforcement learning” OR “computer vision” OR “decision support system” OR “digital twin” OR “digital twins” OR “digital twinning”)
		PubMed	(“artificial intelligence”[Title/Abstract] OR “machine learning”[Title/Abstract] OR “deep learning”[Title/Abstract] OR “neural network”[Title/Abstract] OR “large language model”[Title/Abstract] OR “generative ai”[Title/Abstract] OR “reinforcement learning”[Title/Abstract] OR “computer vision”[Title/Abstract] OR “decision support system”[Title/Abstract] OR “digital twin”[Title/Abstract] OR “digital twins”[Title/Abstract] OR “digital twinning”[Title/Abstract])
Multi-omics	Integrated biological data layers including genomics, epigenomics, transcriptomics, proteomics, metabolomics, and microbiome research.	OpenAlex	(“multi-omics” OR multiomics OR “multi-omic” OR multiomic OR genomics OR genome OR genetic OR genetics OR transcriptomics OR transcriptome OR proteomics OR proteome OR metabolomics OR metabolome OR epigenomics OR epigenetics OR microbiome OR “gut microbiome”)
		Web of Science	TS=(“multi-omics” OR multiomics OR “multi-omic” OR multiomic OR genomics OR genome OR genetic OR genetics OR transcriptomics OR transcriptome OR proteomics OR proteome OR metabolomics OR metabolome OR epigenomics OR epigenetics OR microbiome OR “gut microbiome”)
		Scopus	TITLE-ABS-KEY(“multi-omics” OR multiomics OR “multi-omic” OR multiomic OR genomics OR genome OR genetic OR genetics OR transcriptomics OR transcriptome OR proteomics OR proteome OR metabolomics OR metabolome OR epigenomics OR epigenetics OR microbiome OR “gut microbiome”)
		PubMed	(“multi-omics”[Title/Abstract] OR multiomics[Title/Abstract] OR “multi-omic”[Title/Abstract] OR multiomic[Title/Abstract] OR genomics[Title/Abstract] OR genome[Title/Abstract] OR genetic[Title/Abstract] OR genetics[Title/Abstract] OR transcriptomics[Title/Abstract] OR transcriptome[Title/Abstract] OR proteomics[Title/Abstract] OR proteome[Title/Abstract] OR metabolomics[Title/Abstract] OR metabolome[Title/Abstract] OR epigenomics[Title/Abstract] OR epigenetics[Title/Abstract] OR microbiome[Title/Abstract] OR “gut microbiome”[Title/Abstract])
Wearable technologies	Wearable sensors and consumer health monitoring devices for physiological and behavioral data acquisition.	OpenAlex	(“wearable technology” OR “wearable technologies” OR “wearable device” OR “wearable devices” OR “wearable sensor” OR “wearable sensors” OR smartwatch OR “smart watch” OR “fitness tracker” OR “activity tracker” OR “smart band” OR wristband OR “wrist-worn device” OR “wearable biosensor”)
		Web of Science	TS=(“wearable technology” OR “wearable technologies” OR “wearable device” OR “wearable devices” OR “wearable sensor” OR “wearable sensors” OR smartwatch OR “smart watch” OR “fitness tracker” OR “activity tracker” OR “smart band” OR wristband OR “wrist-worn device” OR “wearable biosensor”)
		Scopus	TITLE-ABS-KEY(“wearable technology” OR “wearable technologies” OR “wearable device” OR “wearable devices” OR “wearable sensor” OR “wearable sensors” OR smartwatch OR “smart watch” OR “fitness tracker” OR “activity tracker” OR “smart band” OR wristband OR “wrist-worn device” OR “wearable biosensor”)
		PubMed	(“wearable technology”[Title/Abstract] OR “wearable technologies”[Title/Abstract] OR “wearable device”[Title/Abstract] OR “wearable devices”[Title/Abstract] OR “wearable sensor”[Title/Abstract] OR “wearable sensors”[Title/Abstract] OR smartwatch[Title/Abstract] OR “smart watch”[Title/Abstract] OR “fitness tracker”[Title/Abstract] OR “activity tracker”[Title/Abstract] OR “smart band”[Title/Abstract] OR wristband[Title/Abstract] OR “wrist-worn device”[Title/Abstract] OR “wearable biosensor”[Title/Abstract])
Precision exercise	Individualized or personalized exercise interventions and adaptive training approaches.	OpenAlex	(“precision exercise” OR “personalized exercise” OR “personalised exercise” OR “precision sport” OR “personalized training” OR “personalised training” OR “individualized training” OR “individualised training” OR “tailored exercise” OR “tailored training” OR “adaptive exercise” OR “exercise prescription”)
		Web of Science	TS=(“precision exercise” OR “personalized exercise” OR “personalised exercise” OR “precision sport” OR “personalized training” OR “personalised training” OR “individualized training” OR “individualised training” OR “tailored exercise” OR “tailored training” OR “adaptive exercise” OR “exercise prescription”)
		Scopus	TITLE-ABS-KEY(“precision exercise” OR “personalized exercise” OR “personalised exercise” OR “precision sport” OR “personalized training” OR “personalised training” OR “individualized training” OR “individualised training” OR “tailored exercise” OR “tailored training” OR “adaptive exercise” OR “exercise prescription”)
		PubMed	(“precision exercise”[Title/Abstract] OR “personalized exercise”[Title/Abstract] OR “personalised exercise”[Title/Abstract] OR “precision sport”[Title/Abstract] OR “personalized training”[Title/Abstract] OR “personalised training”[Title/Abstract] OR “individualized training”[Title/Abstract] OR “individualised training”[Title/Abstract] OR “tailored exercise”[Title/Abstract] OR “tailored training”[Title/Abstract] OR “adaptive exercise”[Title/Abstract] OR “exercise prescription”[Title/Abstract])

OpenAlex was used to capture broad cross-disciplinary developments, PubMed to reflect trajectories within biomedical and health-related research, and Web of Science together with Scopus to provide cross-database validation under different indexing systems and disciplinary structures. For each database–domain combination, the annual number of records returned by the corresponding query was used as the unit of analysis. Publication counts were retained and analysed separately for each database rather than pooled across sources, because differences in database coverage and indexing structure preclude direct aggregation. Annual publication counts are summarized in **Supplementary Table 1**.

Absolute annual publication counts were first plotted to describe the scale and temporal development of each domain. Because large differences in publication volume could obscure comparisons of relative growth trajectories, the annual counts for each domain were additionally normalized to the maximum count observed for that domain within the same database during 2010–2025, according to the following equation:


$${\text{S}}_{\text{d},\text{ k},\text{ t}}=\frac{{\text{N}}_{\text{d},\text{ k},\text{ t}}}{{\text{max}}_{\text{t}\in \{2010,\;\ldots ,\;2025\}}{\text{N}}_{\text{d},\text{ k},\text{ t}}}$$

where N(*d, k, t*) denotes the number of publications indexed in database *d* for domain *k* in year *t*, and S(*d, k, t*) denotes the corresponding normalized publication count. A value of 1 therefore represents the peak annual publication count for a given domain within a given database, whereas values below 1 indicate the proportion of that peak observed in other years. This transformation was intended to facilitate comparison of temporal trajectories rather than absolute publication volumes. The analysis was descriptive, and the reproducibility of the observed patterns was assessed by examining their consistency across the four databases.

Across the databases, all four domains exhibited substantial long-term growth between 2010 and 2025, despite some domain- and database-specific short-term fluctuations. Artificial intelligence showed the largest absolute expansion and substantially exceeded the other domains in publication volume. Multi-omics displayed comparatively stable and continuous development, whereas wearable technologies and precision exercise, despite their smaller initial publication bases, demonstrated pronounced relative increases characteristic of emerging application-oriented fields (**Figure 2**; **Supplementary Table 1**). These broad patterns were reproduced across OpenAlex, PubMed, Web of Science, and Scopus, although the magnitude and timing of individual changes varied according to database coverage and indexing structure. The cross-database consistency therefore supports interpreting the observed trajectories as broader developments in the research landscape rather than as patterns attributable solely to a single database.

The normalized series showed a broadly synchronized acceleration after approximately 2018, with particularly steep later-stage growth in artificial intelligence and wearable technologies (**Figure 2B**, 1D, 1F, 1H). Taken together, these findings suggest that the constituent technologies of precision exercise have developed in parallel and are displaying increasingly aligned growth trajectories. Against this background, an increasingly important methodological challenge is how these technologies can be systematically integrated into a stable, maintainable, and scalable closed-loop system capable of supporting day-scale decision-making across diverse populations and real-world scenarios. The following sections therefore examine the functional roles and practical boundaries of wearable devices, multi-omics, and artificial intelligence within precision exercise, before introducing the WMRE2030 methodological integration framework.

## 4 Evolution and unresolved gaps in related frameworks: the methodological contribution of WMRE2030

Each turn in the evolution of related frameworks was driven by practical problems that remained unresolved at the time, with the principal trajectory summarized in **Table 2**. Initially, the Exercise is Medicine initiative brought physical activity from general health advice into clinical practice, making it an intervention that could be assessed, prescribed, and referred to appropriate professionals (Cowan, 2016). As systems biology, multi-omics, and medical digitalization broadened the scope of health management, P4 systems medicine shifted the focus from reactive management after disease onset toward predictive, preventive, personalized, and participatory management (Hood and Flores, 2012); the population perspective of precision medicine further incorporated feasibility, equity, and overall health benefits (Khoury et al., 2012), while Exercise as Personalized Medicine introduced this concept into exercise science, positioning response heterogeneity, dose optimization, and subgroup differences as important starting points for precision exercise (Buford et al., 2013). With mobile devices and continuous sensing technologies entering everyday life, the question expanded from “what should be delivered” to “when, according to which state, and how it should be delivered.” The Behavioral Intervention Technology model accordingly linked intervention goals, behavioral strategies, technological components, and workflows (Mohr et al., 2014), whereas JITAI embedded decision points, tailoring variables, and decision rules within real-world contexts, enabling interventions to adapt dynamically to individual states (Nahum-Shani et al., 2015, Nahum-Shani et al., 2018). Meanwhile, precision exercise medicine began to distinguish true response variability from measurement error and to examine its biological modifiers (Ross et al., 2019), while decision-support frameworks in high-performance sport sought to transform the growing volume of monitoring data into systematic, transparent, and actionable practical judgments (Schelling and Robertson, 2020). In recent years, this pathway has continued toward dynamic representation, knowledge interoperability, and trustworthy intelligence. Digital twins for health connect real individuals, virtual representations, and continuous data streams, bringing monitoring, simulation, prediction, and intervention testing into the same dynamic process (Hliš et al., 2024; Katsoulakis et al., 2024; Shen et al., 2024); EXMO, in turn, provides a shareable and extensible semantic foundation for fragmented exercise and health knowledge (Liu et al., 2024). At the same time, AI-enabled wearable technologies have begun to move from passive recording toward insight generation, judgment, and decision support (Düking et al., 2025), while the CeHRes Roadmap 2.0 and FUTURE-AI have incorporated contextual adaptation, continuous evaluation, traceability, robustness, explainability, and human oversight into system development and governance (Lekadir et al., 2024; Kip et al., 2025). By 2026, JITAI-Twin had further used digital-twin simulation and feedback from deployment data to continuously optimize adaptive intervention algorithms, marking a shift in related frameworks from static design toward dynamically updated systems (Gazi et al., 2026).

**Table 2:** Historical evolution of representative frameworks and paradigms informing WMRE2030.

Year	Representative framework or paradigm	Historical driver/context^a^	Key methodological advance^b^	References
2007	Exercise is Medicine initiative	Physical inactivity had become a major public health concern, yet exercise had not been systematically incorporated into routine clinical assessment and prescription.	Established a clinical pathway for physical activity assessment, exercise prescription, and referral to exercise professionals.	(Cowan, 2016)
2012	P4 systems medicine and the population perspective of precision medicine	The post-genomic era required a shift from reactive treatment after disease onset toward proactive health management.	Linked predictive, preventive, personalized, and participatory care with systems biology, multi-omics, and digital technologies, while emphasizing the balance between individual precision and population health.	(Hood and Flores, 2012); (Khoury et al., 2012)
2013	Exercise as Personalized Medicine	Personalized medicine remained largely centered on pharmacogenomics, while heterogeneity in exercise-training responses received insufficient attention.	Emphasized analysis of individual responses, sources of response variability, subgroup identification, and optimization of exercise dose.	(Buford et al., 2013)
2014	Behavioral Intervention Technology (BIT) Model	Smartphones, web platforms, and sensors were entering health interventions, but a unified model linking clinical aims to technological implementation was lacking.	Connected intervention goals, behavioral strategies, technological components, and workflows through the questions of why, what, how, and when.	(Mohr et al., 2014)
2015–2018	Just-in-Time Adaptive Intervention (JITAI)	Static interventions were poorly suited to rapidly changing needs and contexts in everyday life.	Established decision points, tailoring variables, intervention options, and decision rules so that support could adapt to changing states and contexts.	(Nahum-Shani et al., 2015, Nahum-Shani et al., 2018)
2019	Precision Exercise Medicine	Population-average effects were insufficient to explain individual responses to the same exercise stimulus.	Systematically addressed true inter-individual response variability, measurement error, response classification, and biological modifiers.	(Ross et al., 2019)
2020	Development framework for decision support systems in high-performance sport	The rapid growth of monitoring data in high-performance sport created information overload, while experience-led decision-making constrained data translation.	Proposed principles for developing and evaluating systems that translate complex sport data into structured decisions.	(Schelling and Robertson, 2020)
2024	Digital twins for health and sport	Single assessments and static models could not continuously represent individual states or adequately predict intervention outcomes.	Introduced real entities, dynamic virtual representations, simulation, prediction, and bidirectional information updating.	(Hliš et al., 2024; Katsoulakis et al., 2024; Shen et al., 2024)
2024	Exercise Medicine Ontology (EXMO)	Fragmented concepts in exercise prescription, health information, and device terminology constrained interoperability across databases and recommendation systems.	Provided a core semantic structure for exercise, health status, testing, and prescription knowledge, with support for population- or disease-specific extensions.	(Liu et al., 2024)
2025	Classification system for AI-enabled wearable automation in exercise decision-making	Wearable devices were progressing from recording tools toward automated judgment, but their degree of automation lacked a common descriptive framework.	Classified system capabilities across data acquisition, structuring, insight generation, judgment, and automated decision-making.	(Düking et al., 2025)
2025	CeHRes Roadmap 2.0	The development, implementation, and evaluation of digital health technologies were often treated as disconnected processes, leading to poor alignment among technologies, users, and implementation contexts.	Emphasized iterative alignment among context, users, technology development, implementation, and continuous evaluation.	(Kip et al., 2025)
2025	FUTURE-AI framework	Although the experimental performance of AI models was improving rapidly, cross-context deployment, trust, safety, and generalizability remained constrained.	Established principles of fairness, universality, traceability, usability, robustness, and explainability.	(Lekadir et al., 2024)
2026	JITAI digital-twin framework (JITAI-Twin)	JITAI algorithms required continual optimization, but repeated real-world deployment was costly and could not efficiently test all design alternatives.	Used digital twins of target subpopulations to simulate decision algorithms and continuously update the simulation environment with deployment data.	(Gazi et al., 2026)

a Historical drivers/contexts are concise syntheses of the problems or contextual conditions described in the cited literature and are not verbatim statements from the original sources. ^b^Key methodological advances summarize the principal contribution of each framework or paradigm rather than its full scope or subsequent development. AI, artificial intelligence; BIT, Behavioral Intervention Technology; EXMO, Exercise Medicine Ontology; JITAI, Just-in-Time Adaptive Intervention; P4, predictive, preventive, personalized, and participatory.

However, as these frameworks have continued to evolve, a deeper methodological gap has become increasingly apparent: although existing frameworks separately provide capabilities for state monitoring, mechanistic interpretation, dynamic prediction, or decision support, they remain fragmented and have not yet formed a day-scale closed loop that integrates high-frequency state fluctuations, biological information acquired and updated at data-type-specific timescales, exercise-prescription execution, and real-world response feedback, while operating across clinical populations, the general population, and high-performance athletes. Compared with JITAI, which primarily focuses on the timing of behavioral prompts; digital twins, which emphasize dynamic individual representation; precision exercise medicine, which centers on explaining response heterogeneity; and AI frameworks, which focus on algorithmic capability or governance principles, WMRE2030 further operationalizes these components within an architecture that uses the day scale as the core decision level and places exercise execution and response updating at its center. Its true novelty lies not in the individual technological elements that constitute the framework, but in the cross-population design principle of “the same architecture, different parameters”: clinical patients, generally healthy individuals, and high-performance athletes can share the same stable closed-loop structure of state sensing–constraint-based interpretation–decision support–exercise execution–response feedback, while using different sensors, omics inputs, AI models, decision thresholds, and levels of oversight according to their respective health risks, exercise goals, and contexts of use. Accordingly, WMRE2030 is positioned as a precision-exercise methodological framework centered on the day scale, capable of cross-population adaptation, and parameterizable according to specific contexts. At the same time, it provides a “methodological toolbox” that future studies can flexibly draw upon: according to the health status, exercise goals, risk level, and data availability of clinical patients, the general population, or high-performance athletes, researchers can select the corresponding wearable devices, multi-omics indicators, artificial intelligence models, decision thresholds, and oversight mechanisms, and design and test specific interventions within a unified closed-loop logic.

## 5 Usability and boundaries of wearable devices in exercise and health management

### 5.1 Available metrics and typical application scenarios across wearable device locations

Some consumer wearable devices also incorporate irregular-rhythm notification features that have been evaluated through regulatory pathways (FDA, 2018). Consumer-grade wearables can now generate a relatively stable set of metrics relevant to clinical and exercise applications, including step count and activity intensity, heart rate and heart rate variability–related indices, resting heart rate, sleep duration and fragmentation, and, in some devices, blood oxygen saturation, skin temperature trends, and respiratory rate (Petek et al., 2023). Their main advantage lies in enabling long-term, low-burden monitoring across daily life, training, and selected chronic disease contexts. At the same time, many commonly used outputs—particularly sleep staging and some HRV-derived indicators—remain strongly dependent on proprietary algorithms, and therefore require cautious interpretation within clearly defined physiological and contextual boundaries (Petek et al., 2023).

In exercise monitoring, device location is closely linked to the type of signal that can be captured and the scenario in which it is most informative. From a practical perspective, wearable devices can be broadly understood as targeting either internal load or external load, with different body locations offering distinct capabilities (Seshadri et al., 2019). Wrist- and chest-worn devices are primarily used for continuous cardiovascular and recovery-related monitoring, whereas trunk- or back-mounted systems integrating GPS and inertial sensors are especially valuable for external-load quantification in team-sport settings (Bourdon et al., 2017). Finger-worn smart rings, owing to their relatively stable nocturnal signal acquisition, have emerged as representative devices for monitoring sleep, recovery, and circadian patterns (Gong et al., 2025). Foot-based smart insoles are primarily applied in gait analysis, running training, and rehabilitation, with plantar pressure and spatiotemporal parameters systematically summarized in multiple reviews (Santos et al., 2024). Meanwhile, smart garments based on conductive textiles show potential for respiratory, postural, and multimodal physiological monitoring, although limitations in durability and lack of standardization still constrain large-scale deployment (Angelucci et al., 2021; Rotzler et al., 2021). Skin-adherent ECG patches, on the other hand, are increasingly used in clinical and research settings for continuous, high-quality electrocardiographic monitoring and have demonstrated clear value in screening high-risk populations (Steinhubl et al., 2018).

Based on the above evidence and application landscape, the present study provides a structured synthesis of mainstream wearable devices according to the framework of “device location—available metrics—typical application scenarios” (**Table 3**). This approach aims to more clearly delineate the capability boundaries of the input layer under conditions of high device heterogeneity, and to provide a reference framework for subsequent discussions on data quality, user adherence, interpretability boundaries, and research applications.

**Table 3:** Available metrics, strengths, limitations, and target populations/scenarios of wearable devices according to anatomical wearing location.

Wearing location	Device form	Available metrics	Main advantages	Main limitations	Target populations/scenarios	References
Wrist	Smartwatches, Fitness Bands	Activity (steps, duration, intensity); Heart rate (PPG); Some HRV; Resting heart rate; Sleep (duration, interruptions, stages—algorithm-based); Some: SpO_2_, skin/temperature trends, respiratory rate, GPS	High wear time compliance; Continuous data; Broad metric coverage; Mature ecosystem, suitable for long-term follow-up	Exercise-related noise affecting HR/HRV; Grip-dominant or isometric upper-limb exercise (e.g., weightlifting or rowing) may reduce wrist-PPG accuracy through altered local perfusion and increased motion artefacts;Algorithmic black-box, cross-brand comparability limited; Wear position and tightness affect signal quality; Medical features often serve as alerts	General population; Daily activity and lifestyle management; Basic training monitoring; Daily monitoring for some chronic disease populations;Use cautiously for HR/HRV monitoring during grip-intensive exercise; chest-strap ECG may be preferable when greater accuracy is required.	(FDA, 2018; Perez et al., 2019; Hoog Antink et al., 2021; Petek et al., 2023; Ding et al., 2024; Frodi et al., 2024; Köhler et al., 2024; Dial et al., 2025; Schweizer and Gilgen-Ammann, 2025; Schyvens et al., 2025)
Upper Arm	Heart rate arm strap	Heart rate (more stable during exercise); Training zone (HR zone/intensity distribution)	More stable heart rate signal than wrist-based devices during exercise; More comfortable than chest straps	Limited metric variety (mainly HR/zones)	Running/fitness scenarios for heart rate monitoring; Alternative input when wrist-based HR is unstable	(Yu et al., 2022; Liu et al., 2025b; Schweizer and Gilgen-Ammann, 2025)
Finger	Smart rings	Nighttime heart rate; Nighttime HRV; Sleep duration, interruptions, stages; Temperature/skin temperature trends; Baseline activity	High signal quality at night; Minimal exercise-induced noise; Discreet and comfortable wear; Suitable for recovery and circadian rhythm assessment	Limited ability to capture daytime activity load; Sensitive to finger size and wear position	Healthy population focused on recovery and sleep; Supplementary monitoring for high-level athletes’ recovery	(De Zambotti et al., 2019; Cao et al., 2022; Kristiansson et al., 2023; Svensson et al., 2024; Gong et al., 2025)
Chest	Heart rate chest strap	Heart rate (high accuracy); HRV; Heart rate zones	Resistant to exercise-induced noise; Highest heart rate accuracy during high-intensity exercise	Not suitable for all-day wear; Lower comfort and compliance; Limited metric variety	Athletes; High-intensity training and competition monitoring; Research testing	(Pasadyn et al., 2019; Cosoli et al., 2020; Marzano-Felisatti et al., 2024; MaChado et al., 2025)
Trunk/Back	Vest-style GPS/IMU	External load: Distance, speed zones, sprint counts, acceleration/deceleration, direction change; Some heart rate integration	Most effective for monitoring external load in team sports; Supports training load management	High cost; Closed systems; High data interpretation threshold; Non-comparable across systems	Professional or high-level team sports; Strength and conditioning coaches for load management	(Beato et al., 2018; Delves et al., 2021; Torres-Ronda et al., 2022; Ferraz et al., 2023; Dawson et al., 2026)
Skin-Adherent	ECG patches, respiration/temperature patches, CGM patches	Continuous ECG; Heart rate/HRV; Respiratory rate; Temperature trends; Posture/activity; (CGM) continuous glucose curve, variability and derived metrics	Strong continuity; High signal quality; Close to medical-grade monitoring	High cost and operational complexity; Skin irritation; Not suitable for long-term use by general population	Clinical and research monitoring; High-risk populations; Wrist-worn data validation	(Breteler et al., 2018; Steinhubl et al., 2018; Downey et al., 2019; Choi et al., 2020; Muñoz Fabra et al., 2021; Diabetes Care, 2025)
Wrist (Cuff-style)	Wearable blood pressure devices	Systolic/diastolic pressure (intermittent); Pulse rate	Direct blood pressure readings; Suitable for home and follow-up management	Non-continuous; Requires specific measurement posture and conditions; Large wearable volume	Hypertension or high-risk populations; Home monitoring	(Khoshdel, 2010; Kuwabara et al., 2019; Mancia et al., 2023)
Foot	Smart insoles, Foot sensors	Plantar pressure; Contact time; Gait parameters; Left-right symmetry	Direct reflection of gait and impact characteristics; Related to injury risk	Dependent on shoe type; Wear and algorithmic differences; Costly	Running training; Rehabilitation; Gait evaluation	(Cramer et al., 2022; Elstub et al., 2022; Mason et al., 2023; Santos et al., 2024)
Clothing	Smart clothing, conductive textiles	Respiration/chest movement; Posture; Some electromyography/heart rate	Large-area contact; Suitable for respiratory and posture monitoring	Issues with laundering and durability; Lack of standardization; Limited commercial maturity	Rehabilitation, research, high-level training (exploratory)	(Arquilla et al., 2020; Angelucci et al., 2021; Rotzler et al., 2021; Hussain et al., 2023; Lee et al., 2025)
Multiple Locations	Disease-specific wearables	EDA, accelerometer, heart rate, skin temperature; Event risk alerts	Optimized for specific disease tasks; Capable of event alerts	Narrow applicability; Complex false/near-miss alert management; Relies on care systems	Specific disease populations; Caregiving and clinical research	(Regalia et al., 2019; Hubbard et al., 2021; Onorati et al., 2021; Faust et al., 2025)

PPG, photoplethysmography, a method used for optical heart rate monitoring based on blood volume changes; HR, heart rate; HRV, heart rate variability, reflecting autonomic nervous system regulation; SpO_2_, peripheral capillary oxygen saturation (optically estimated); ECG, electrocardiography signal; CGM, continuous glucose monitoring system; GPS, global positioning system, used for estimating distance, speed, and displacement; IMU, inertial measurement unit, typically including accelerometers, gyroscopes, and magnetometers; EDA, electrodermal activity, reflecting sympathetic nervous system–related skin conductance changes.6 The potential and necessity of multi-omics as a biological “explanatory layer”.

### 5.2 Evolution of wearable device measurement capabilities and contextual applicability boundaries

Given the context-dependent nature of wearable device data quality and interpretability, the discussion surrounding the “reliability” of wearable data has evolved from simple accuracy comparisons to a more systematic delineation of their applicable boundaries and usable conditions. Previous systematic reviews have shown that, under static or low-intensity conditions, most devices provide acceptable accuracy for metrics such as heart rate and activity-related indices. However, under dynamic or high-intensity exercise scenarios, the error margin increases, and cross-device consistency is relatively limited (Fuller et al., 2020b). Subsequent comparative studies have further confirmed that such inconsistencies are highly context-dependent. Even within the same optical pulse transit time (PPG) technology, device consistency with ECG reference signals remains highly dependent on measurement location, activity type, and exercise intensity, making devices difficult to interchange without constraints (Alfonso et al., 2022). In this context, the INTERLIVE Network proposed expert consensus guidelines for evaluating the effectiveness of consumer-grade wearable devices, emphasizing that validation studies must strictly define testing scenarios, reference standards, and statistical methods to avoid generalizing validation results from specific conditions to mismatched contexts (Mühlen et al., 2021). This shift marks a transition from questions of “whether devices are accurate” to inquiries into “under which conditions are they usable.” In terms of metric interpretation, heart rate variability (HRV) and pulse rate variability (PRV), derived from PPG, have become central to ongoing discussions. Recent evidence suggests that the consistency between PRV and ECG-derived HRV across commonly used time-domain and frequency-domain indices is limited, and discrepancies tend to increase with changes in activity state (Kantrowitz et al., 2025). ECG-derived HRV is typically calculated from successive R–R intervals and reflects beat-to-beat variability in cardiac electrical activity (Shaffer and Ginsberg, 2017). In contrast, PRV is calculated from peripheral pulse-wave intervals detected using PPG and provides an indirect estimate of heart-rate dynamics (Gil et al., 2010). Because pulse-wave propagation from the heart to a peripheral measurement site is influenced by the pre-ejection period, pulse transit time, vascular tone, arterial compliance, blood pressure, and peripheral perfusion, PRV contains information about cardiac rhythm while also being modulated by peripheral vascular characteristics (Payne et al., 2006; Allen, 2007; Mejía-Mejía et al., 2020; Yuda et al., 2020). In addition, measurement site, body posture, and local vascular-bed characteristics may affect the agreement between PRV and ECG-derived HRV (Yuda et al., 2020). During physical activity or psychological stress, rapidly changing haemodynamic conditions and instability in PPG fiducial-point detection may further increase the divergence between the two measures (Gil et al., 2010; Mejía-Mejía et al., 2020; Singstad et al., 2021). Therefore, although PRV and HRV may show high agreement under some resting and strictly controlled conditions, PRV should not be regarded as a biologically interchangeable surrogate for ECG-derived HRV during physical activity or exercise (Mejía-Mejía et al., 2020; Yuda et al., 2020; Singstad et al., 2021). Nevertheless, when data acquisition is standardized, signal quality is adequate, and analytical procedures are appropriate, PRV may still serve as a context-specific relative indicator of trends in autonomic regulation (Gil et al., 2010; Mejía-Mejía et al., 2020). Clearly distinguishing between the two measures helps identify limitations in signal quality and sources of error, while clarifying the boundaries among raw signals, algorithmically derived metrics, and physiological constructs.

Beyond signal quality, adherence to device use is a crucial practical constraint determining the usability of wearable data. Unlike traditional laboratory measurements, which are controlled, real-world wearable usage can be influenced by factors such as wear time, device interruptions due to charging, and device replacement. Recent studies have quantified wear time and adherence days as measurable, reportable variables, demonstrating that, with multi-month follow-ups, most populations can maintain relatively high long-term wearability. However, individual behavior differences and device interruptions remain significant influences (Ding et al., 2024; Frodi et al., 2024). Overall, the data quality and interpretability boundaries of wearable devices are not insurmountable technical barriers but rather a series of practical constraints that need to be converged through real-world contexts. This evolutionary trend provides a feasible foundation for wearable devices to function within a structured, day-scale decision-support system and lays the methodological groundwork for embedding wearable data into continuous feedback loops.

### 5.3 Advantages of wearable devices as input layer in day-scale closed-loop systems

Compared to traditional questionnaires or recall-based self-reporting, the core advantage of wearable devices lies in their continuous, low-burden, and objective nature, enabling long-term recording of behavioral and physiological trajectories in real-world settings, significantly reducing recall bias and social desirability-induced systemic errors. Previous research has shown that there is limited consistency between self-reports and objective measurements for key behavioral indicators such as physical activity, with high individual-level variability (Prince et al., 2008). In contrast, real-time or near-real-time sampling is more effective at capturing day-to-day fluctuations and context-dependent changes, greatly enhancing ecological validity (Shiffman et al., 2008). Therefore, in closed-loop systems that require continuous updating of individual states at a “day scale, “ wearable devices are better suited as the input layer, while questionnaires are more appropriate for providing low-frequency background information.

From a methodological standpoint, day-scale closed loops are not simply about high-frequency measurements but represent a structured dynamic intervention paradigm. Just-in-Time Adaptive Interventions (JITAI) break down the intervention process into decision points, individualized adjustment variables, and decision rules, with the premise that there exists a continuous input stream to support real-time decisions regarding “when, to whom, and what support to provide” (Nahum-Shani et al., 2018; Fuller et al., 2020b). Correspondingly, Micro-Randomized Trials (MRT) randomize intervention options at multiple decision points to estimate proximal effects and optimize decision rules, making them the core evaluation tool for JITAI research (Klasnja et al., 2015; Golbus et al., 2021; Qian et al., 2021). Real-world studies have further validated this logic. Research based on MRT has shown that push notifications for sleep feedback triggered by objective monitoring can improve sleep behavior stability on a day scale, providing direct evidence for the “continuous perception—real-time feedback—behavioral adjustment” closed-loop mechanism (Takeuchi et al., 2022, Takeuchi et al., 2024).Overall, the role of wearable devices as the core input layer of a day-scale closed-loop system has gradually emerged through the continuous convergence of theoretical and empirical evidence from JITAI, MRT, and real-world digital interventions. As continuous sensing, contextual recognition, and dynamic feedback have been repeatedly demonstrated as essential components for enabling individualized interventions, wearable devices have correspondingly become a critical foundation for operationalizing such closed-loop systems due to their objective, low-burden, and sustainable data acquisition capabilities.

### 6.1 The role of multi-omics in explaining pathways of inter-individual variability

Substantial inter-individual variability in exercise capacity, training adaptation, metabolic phenotypes, and health risk has long been attributed to the combined effects of “genetic background” and “environmental factors.” However, this dichotomous formulation provides limited insight into the actual mechanisms underlying the emergence of such variability. Accumulating evidence consistently indicates that external environmental and behavioural factors do not act directly on phenotypes; rather, they are progressively translated into functional states and adaptive outcomes through multiple biological layers, including gene regulation, epigenetic modification, transcription, protein expression, and metabolism.

Within this process, epigenetics is widely regarded as a key interface linking the genome with environmental exposures. Environmental stimuli can modulate gene accessibility and transcriptional potential, thereby influencing downstream molecular pathways and phenotypic expression (Bell and Beck, 2010). Intervention studies employing multi-omics approaches in humans have provided direct evidence supporting this mechanism, demonstrating that exercise stimuli can induce epigenetic reprogramming coupled with transcriptional changes, thereby contributing to inter-individual differences in training responses (Lindholm et al., 2014).

This mechanistic understanding has been further extended at the systems level. The Molecular Transducers of Physical Activity Consortium (MoTrPAC) has proposed that, from a systems biology perspective, exercise represents a physiological perturbation whose effects are propagated through multi-layered molecular networks across tissues and time scales before manifesting as functional adaptations (Sanford et al., 2020; Jakicic et al., 2024). Longitudinal personal omics studies, along with exposome–multi-omics integration frameworks, have further demonstrated that environmental exposures and life events leave traceable dynamic signatures across multiple molecular phenotypes, facilitating a transition from descriptive phenotyping toward biologically grounded subtype characterization (Chen et al., 2012; Miller, 2021; Gao et al., 2022; Baccarelli et al., 2023).

From a systems biology standpoint, single-omics approaches are insufficient to capture the complexity of biological processes. Only through integrated multi-omics analysis can consistent, cross-layer mechanistic explanations be achieved (Subramanian et al., 2020). Therefore, the core value of multi-omics does not lie in directly generating exercise prescriptions or predicting the outcomes of single interventions. Rather, it functions as a biological explanatory layer that helps to open the “black box” linking environment, organism, and response, thereby providing a biologically constrained and interpretable pathway for understanding inter-individual variability.

### 6.2 Cost democratization and real-world application window

Within real-world service and research systems, different omics layers vary substantially in accessibility, maturity, and methodological role (**Figure 2**; **Supplementary Table 2**). Among them, genomics is currently the most mature and widely accessible. The declining costs of sequencing and genotyping have made genetic information increasingly obtainable at scale, supporting its use in stratified assessment of exercise responsiveness, nutritional and metabolic traits, sleep and circadian tendencies, and disease susceptibility (NHGRI, 2023). At the same time, systematic reviews and policy analyses have consistently highlighted concerns regarding misinformation, privacy, regulation, and over-interpretation in direct-to-consumer contexts (Martins et al., 2022; Bujang et al., 2025; Hull et al., 2025). Accordingly, in precision exercise systems, genomic information is better positioned as a relatively stable background layer that supports long-term stratification, rather than as a stand-alone basis for individualized prescription.

Epigenetic markers and the gut microbiome occupy a more intermediate position. Both can capture aspects of long-term lifestyle, environmental exposure, and metabolic state, and therefore hold value for biological characterization beyond static genotypes. However, their real-world usability remains constrained by interpretability, standardization, and cross-platform consistency (Apsley et al., 2025; Hauskeller and Shore, 2025)- (Hoffmann et al., 2024; Pichon et al., 2025; Porcari et al., 2025). Current evidence suggests that these layers are more appropriate for low-frequency calibration, contextual enrichment, and hypothesis generation than for direct decision-making at the individual level.

Metabolomics appears to be entering a more deployable phase. Recent evidence suggests that it has moved beyond purely exploratory use toward larger-scale, increasingly standardized applications in cohort studies and risk stratification (Hajjar et al., 2023; Zhang et al., 2024b). By contrast, transcriptomics and proteomics, although highly informative for pathways related to inflammation, immunity, and training adaptation, remain more strongly anchored to research and validation settings because of sample complexity, cost, and limited interpretative stability in routine use (Cui et al., 2024; Dagher et al., 2025). Other specialized omics, including lipidomics, phosphoproteomics, glycomics, and single-cell or spatial omics, were not conceptually excluded but were not discussed in detail because their current applications remain largely mechanistic and research-oriented, with cost, technical complexity, and limited standardization constraining near-term integration into scalable precision-exercise services (Rojas-Macias et al., 2019; Vvedenskaya et al., 2022; Wu et al., 2023; Dezem et al., 2024). This review therefore prioritizes omics layers with greater real-world accessibility and implementation readiness, while acknowledging that these specialized approaches may become increasingly relevant as costs decline and methodological standards mature. Overall, the core value of multi-omics lies in providing explanations and calibration as the individual background layer and risk constraint layer, rather than directly generating exercise prescriptions.

### 6.3 The biological explanatory layer as a critical anchor for the reliability of precision exercise

In the current intelligent exercise systems, wearable devices and artificial intelligence enable personalized exercise recommendations to be rapidly generated on a daily scale based on continuous monitoring data. However, if this closed-loop is solely based on statistical mapping between physiological signals and prescription outputs, without the structural constraints of a biological explanatory layer, its so-called “precision” often remains limited to the learning and reuse of past experiential patterns (Albers et al., 2023; Omar et al., 2023; Gu et al., 2025). It is difficult to achieve genuine individual mechanistic differentiation, thus hindering the long-term reliability and extrapolation of one-to-one exercise decisions (Ghassemi et al., 2021; Cross et al., 2024).

In the absence of mechanistic information, algorithms do not truly understand the biological significance of training stimuli in different individuals. As a result, they are unable to determine which interventions are effective for whom, which are ineffective, and are also unable to recognize when physiological safety boundaries are being approachedLiu et al., 2024. The resulting issue is not prediction error, but rather the algorithm’s lack of “boundary awareness” at the biological level. It cannot differentiate structural differences between individuals in genetic backgrounds, metabolic pathways, inflammatory regulation, and recovery capacity, and such differences will be continuously amplified in long-term, automated, individualized decision-making processes (Cross et al., 2024; Nasb et al., 2025).

In this sense, multi-omics does not aim to provide high-frequency, real-time feedback similar to wearable devices. Its application is often constrained by sampling invasiveness, technical complexity, and cost factors. However, its key advantage lies in serving as the biological explanatory layer within the precision exercise system, providing mechanistic anchors and safety boundaries for algorithms, thus allowing exercise prescriptions to be examined within a clearly defined biological applicability range (Sanford et al., 2020; MoTrPAC Study Group et al., 2024). Therefore, the core value of multi-omics lies in providing periodic, low-frequency mechanistic calibration and risk delineation for intelligent systems, enabling the intelligent exercise system to evolve from a “signal-driven closed loop” to a “mechanism-constrained closed loop, “ thereby supporting the long-term reliable operation of precision exercise in the real world (Vallée, 2024).

At the operational level, multi-omics and wearable data do not need to be updated synchronously or at the same frequency. Instead, an asynchronous, multi-frequency, and hierarchically integrated acquisition strategy should be adopted according to the biological stability, rate of change, sampling invasiveness, testing cost, analytical latency, and practical decision-making requirements of each data type. Specifically: 1) genomic information is relatively stable and can generally be assessed once at baseline to serve as the individual’s long-term biological background; 2) dynamic omics layers, including the epigenome, transcriptome, proteome, metabolome, and microbiome, should be reassessed periodically or in response to predefined events according to their temporal sensitivity and intended application, thereby avoiding the long-term static use of a single measurement while reducing unnecessary sampling burden; and 3) for clearly defined, risk-relevant biomarkers that can be obtained through low-burden sampling or continuous monitoring devices, the acquisition frequency may be increased to daily, high-frequency, or continuous measurement according to disease risk and intervention needs, as exemplified by glucose monitoring in individuals with diabetes. Complementing these data streams, wearable devices are primarily used to continuously capture high-frequency fluctuations in the phenome, thereby providing information on short-term state changes that multi-omics measurements cannot readily reflect in real time. These different data sources enter the artificial-intelligence decision layer as independent but functionally complementary information streams: multi-omics data provide relatively stable or periodically updated mechanistic context, response potential, and safety boundaries, whereas wearable data provide real-time or near-real-time information on dynamic state changes. Artificial intelligence can therefore integrate information, calibrate models, and update exercise prescriptions across different temporal scales, thereby improving the responsiveness of the day-scale closed loop to changes in individual state while preserving biological interpretability and risk constraints, and ultimately balancing timeliness, precision, and practical feasibility.

## 7 Artificial intelligence as the computational and decision-making hub in precision exercise

### 7.1 The role of artificial intelligence in data integration and decision support in precision exercise

As wearable devices and multi-omics technologies become increasingly accessible, the focus of precision exercise research is shifting from data acquisition itself to how to integrate physiologic and behavioral information from diverse sources with different time scales and transform it into executable, traceable, and physiologically safe decision support structures (Muse and Topol, 2024; Ooka, 2025). In this context, artificial intelligence (AI) serves as the computational and decision-making hub, unifying continuous monitoring data, low-frequency biological background information, and real-time contextual data to support individualized decision-making on a daily scale.

Functionally, AI plays a role in information integration and decision organization within precision exercise. The high heterogeneity of multi-source physiological and behavioral data, coupled with the multi-objective trade-offs required in exercise interventions—such as effectiveness, recovery, compliance, and health risks—necessitates that decisions be contextualized within clear physiological constraints. AI, in this setting, provides structured synthesis and ranking of multidimensional data to support reviewable and executable decision-making plans (Muse and Topol, 2024). It is important to note that this positioning of AI as a “decision hub, not a decision replacement” has been gradually supported in fields such as precision nutrition and medicine. For example, in cardio-metabolic health, studies show that significant heterogeneity in individuals’ metabolic phenotypes, risk backgrounds, and intervention responses makes “one-size-fits-all” lifestyle recommendations ineffective. Instead, multi-dimensional information such as genetics/omics, metabolism, and behavior must be integrated, and AI is primarily used to support risk stratification and intervention decisions, rather than directly generating standardized protocols (Muse and Topol, 2024; Guasch-Ferré et al., 2025). Further research indicates that digital twin-driven personalized health intervention frameworks can continuously absorb multi-source physiological information and dynamically update decision-making within the constraints of physiological mechanisms. In this context, AI serves as the core component linking the data layer and decision layer, supporting state characterization, response prediction, and prospective decision-making (Li et al., 2025; Ooka, 2025; Su et al., 2025).

When AI is embedded in intervention processes and may influence an individual’s daily training or recovery decisions, its reliability cannot be measured solely by predictive performance. It must be assessed based on its clear system role, traceable decision pathways, and well-defined risk constraints. Research reports, regulatory frameworks, and governance guidelines consistently emphasize the necessity of transparent descriptions, human supervision, and accountability to ensure that AI operates within controllable and safe boundaries (Liu et al., 2020).

### 7.2 Implementation pathways of artificial intelligence in precision exercise

From an implementation perspective, the computational role of artificial intelligence in precision exercise should not be understood as a single technological solution, but as a staged continuum ranging from rule-based systems to adaptive learning architectures (**Table 4**). These pathways are not competing endpoints; rather, they represent different levels of complexity, autonomy, and risk, which should be selected according to application context, resource availability, and safety requirements. At the entry level, rule-based systems grounded in guidelines, expert consensus, and predefined thresholds remain the most interpretable and auditable option, particularly in low-risk or safety-sensitive settings.

**Table 4:** Major implementation pathways of artificial intelligence as the computational decision core in precision exercise.

Implementation pathway	Technical characteristics	Strengths	Limitations	Suitable stage/scenario	References
Guideline/Expert rules (Rule-based systems)	Encode guidelines, consensus statements, and expert knowledge into “threshold + condition + rule chain” (if–then), producing traceable rule activation paths	Highly interpretable and auditable; safety thresholds and contraindications easily embedded; low deployment cost	Limited ability to capture complex heterogeneity; low adaptability; maintenance cost increases with system expansion	Early-stage/MVP; public health; chronic disease management with “safety-first” priority; standardized institutional workflows	(Sutton et al., 2020)
Rules + traditional ML (risk stratification/state recognition)	Apply statistical/ML models for classification, regression, or clustering (e.g., risk stratification, anomaly detection), with outputs gated by rule-based hard constraints	Improves personalization under controlled conditions; integrates with rule systems; optional interpretability (e.g., feature importance)	Dependent on data quality and external validation; requires monitoring of model drift; interpretability may still be limited	From MVP to scaling stage; data-rich environments; scenarios requiring finer population stratification	(Sutton et al., 2020)
Prompt engineering	No parameter updates; uses prompt templates and stepwise instructions to organize, summarize, and structure information	Lowest cost and fastest iteration; suitable for transforming multi-source data into structured decision summaries	Stability depends on prompt design; prone to hallucination or factual errors; requires external constraints and verification	Rapid prototyping; auxiliary analysis; “second review” scenarios for coaches, clinicians, or researchers	(Vaid et al., 2024)
Retrieval-Augmented Generation (RAG)	Retrieves external knowledge bases, guidelines, or evidence prior to generation; outputs are grounded in retrieved content	Significantly reduces hallucination; outputs are traceable to evidence; facilitates hard constraints and auditing	Dependent on knowledge base quality and updating; higher system complexity; still requires safety rules and human oversight	Core architecture for deployable systems; guideline-driven personalized recommendations; evidence-referenced decision support	(Lewis et al., 2020)
Domain/Instruction tuning (SFT/Instruction tuning)	Fine-tunes models using domain-specific data and instruction–response pairs to improve task alignment and professional consistency	Enhances consistency and task alignment; better suited for specialized domains	Requires high-quality datasets and evaluation; high maintenance cost; still needs external constraints for safety	Specialized/high-demand scenarios; specific populations or exercise tasks; institutional or research deployments	(Ziegler et al., 2020)
Contextual bandit/Reinforcement learning (online policy learning)	Optimizes “when, for whom, and what intervention” through feedback across repeated decision points; suitable for daily closed-loop adaptation	Learns individual responses and context dependency; suitable for closed-loop optimization	Exploration introduces risk; high requirements for safety constraints and evaluation design; highest deployment barrier	Controlled studies/high-expertise settings; strategy optimization (e.g., within JITAI/MRT frameworks)	(Tewari and Murphy, 2017)
Agent-based systems (Autonomous agents)	LLM-based systems capable of perception–reasoning–action; can call tools (databases, models, rule systems) to complete complex task chains	Enables automation of complex decision workflows; supports multi-module integration (wearables + omics + AI); mimics real-world coach/clinician decision processes	High system complexity; stability and safety depend on external constraints; evaluation frameworks remain immature	Advanced closed-loop systems; multi-source data integration; automated decision support systems	(Wang et al., 2024; Piccialli et al., 2025)
Multimodal AI	Integrates heterogeneous data (text, time series from wearables, images, physiological signals) into unified representations and models	Closer to real physiological–behavioral systems; enhances perception of complex states; supports cross-modal inference	Challenges in data synchronization and alignment; high training cost; limited interpretability across modalities	Multi-source data integration; digital health platforms; high-level training monitoring	(Baltrušaitis et al., 2018; Shaban-Nejad et al., 2023)
Causal ML/Causal AI	Uses causal graphs (DAGs) and potential outcome frameworks to identify “intervention → outcome” relationships rather than correlations	Improves interpretability and generalizability; supports transportability across populations; reduces spurious associations	High demands on data and assumptions; complex modeling; difficult in high-dimensional settings	Intervention design; individualized prescription; cross-population generalization (core of precision exercise)	(Pearl, 2010; Yazdani and Boerwinkle, 2014)
Digital twin/Simulation-based AI	Constructs dynamic virtual representations of individuals to simulate and evaluate intervention strategies	Enables risk-free optimization of interventions; supports individualized strategy evaluation; links biological mechanisms with behavioral feedback	Extremely high modeling complexity; strong dependence on multi-omics and longitudinal data; validation challenges	Advanced stage; long-term precision exercise management; complex populations (e.g., chronic disease, rehabilitation)	(Fuller et al., 2020a; Laubenbacher et al., 2024; Zhang et al., 2024a)
Federated learning/Privacy-preserving AI	Distributed model training without sharing raw data, enabling cross-institution learning while preserving privacy	Strong data privacy protection; enables multi-center data integration; improves model generalizability	High system complexity; communication cost; issues with model consistency and bias remain	Multi-center studies; healthcare/fitness data platforms; cross-institution collaboration	(McMahan et al., 2017; Kairouz and McMahan, 2021; Tao and Li, 2025)

ML, machine learning; RAG, retrieval-augmented generation; SFT, supervised fine-tuning; Instruction tuning, instruction-based fine-tuning; Bandit, multi-armed bandit; JITAI, just-in-time adaptive intervention; MRT, micro-randomized trial; MVP, minimum viable product; hard constraints, rule- or guideline-based enforced safety boundarie.

(Sutton et al., 2020). In such frameworks, conventional machine-learning models may assist with state recognition or risk stratification, but decision authority is still primarily constrained by explicit rules. This makes hybrid rule-plus-model approaches especially suitable for early deployment, where personalization is needed but uncontrolled model behaviour is unacceptable. With the emergence of large language models, a second implementation layer has become increasingly feasible. Prompt-based orchestration, retrieval-augmented generation, and domain-specific tuning allow AI systems to integrate multi-source information and provide more flexible decision support (Lewis et al., 2020; Ziegler et al., 2020). However, their practical value depends on whether model outputs remain anchored to external evidence, rule systems, and human supervision. In this sense, large language models are better viewed as reasoning and integration interfaces than as autonomous decision-makers. A further step involves adaptive learning systems designed for repeated decision-making within day-scale closed loops. Methods such as contextual bandits and reinforcement learning are theoretically attractive because they can optimize intervention timing, selection, and sequencing based on ongoing individual responses.

(Tewari and Murphy, 2017). Translating these methods into practice, however, requires substantially higher methodological and ethical standards, including carefully constrained exploration, rigorous evaluation designs, and explicit safety boundaries. At present, they are therefore more appropriately positioned within controlled research or highly specialized settings than in routine real-world deployment. At the system level, recent developments increasingly point toward collaborative rather than purely model-centric AI. Agent-based architectures can combine reasoning models with external tools, databases, rule engines, and analytical modules to support multi-step, cross-module decision workflows (Wang et al., 2024; Piccialli et al., 2025). In parallel, multimodal learning, causal inference frameworks, digital twins, and privacy-preserving collaborative modelling further expand the horizon of precision exercise AI by improving state representation, transportability, simulation capacity, and cross-site generalizability (McMahan et al., 2017; Baltrušaitis et al., 2018; Kairouz and McMahan, 2021; Shaban-Nejad et al., 2023; Tao and Li, 2025).Nevertheless, these more advanced forms should be understood as future-oriented extensions of the framework rather than immediate prerequisites for implementation.

### 7.3 Daily-scale closed-loop dynamic decision paradigm and the adaptability of artificial intelligence

In real-world living and training contexts, training load, sleep, psychological stress, illness exposure, and recovery capacity exhibit substantial intra-day and inter-day variability. As a result, both the effectiveness and safety boundaries of exercise prescriptions are inherently dynamic. Against this background, the daily-scale closed-loop paradigm represents a methodological shift: exercise prescriptions are no longer static textual outputs, but dynamic decision systems that evolve with the individual’s state.

Just-In-Time Adaptive Interventions (JITAIs) provide a well-established theoretical and design framework for this transition. JITAIs decompose the intervention process into decision points, tailoring variables, and decision rules, enabling systems to deliver contextually appropriate support as individual states change, thereby avoiding both over- and under-intervention (Nahum-Shani et al., 2018). Complementarily, Micro-Randomized Trials (MRTs) offer a causal inference framework for optimizing and evaluating interventions across multiple daily decision points. By randomizing intervention options at each decision point, MRTs enable estimation of proximal effects and continuous refinement of decision rules (Klasnja et al., 2015).

In practical applications, reinforcement learning–based mobile health interventions have demonstrated the feasibility of using artificial intelligence to optimize both the timing and content of interventions across multiple daily decision points, providing direct evidence for AI as the central component of a daily-scale closed-loop system (Liao et al., 2020). Taken together, current evidence suggests that the primary advantage of AI in precision exercise lies not in autonomous prescription generation, but in its capacity—under constraints imposed by evidence, rules, and safety boundaries—to support a dynamic decision architecture that updates with individual states and improves iteratively across repeated decisions. In this sense, AI offers a methodologically viable and promising pathway for establishing the computational and decision-making core of precision exercise systems.

## 8 From conceptual framework to the real world: implementation challenges and future directions of WMRE2030

### 8.1 Data storage dependence and privacy governance

WMRE2030 integrates continuous wearable data, multi-timescale omics, exercise prescriptions, actual loads, and response feedback to form a long-term longitudinal data system. As high-frequency physiological signals and high-dimensional omics data continue to accumulate, local storage and computing capacity are often insufficient (Santiago-Rodriguez and Hollister, 2021; Ates et al., 2022), thereby necessitating reliance on cloud services, third-party platforms, or vendor interfaces. However, this dependence may also create “platform lock-in” through inconsistent data formats, differences in metric definitions, restricted access to raw data, and untraceable algorithm updates, consequently affecting data sharing, model transfer, and research reproducibility (Woolley et al., 2019; Canali et al., 2022; Schumann and Doherty, 2024). Future work should fully document device and algorithm versions, sampling frequencies, timestamps, and preprocessing procedures (Woolley et al., 2019), adhere to the FAIR principles, and promote cross-platform mapping through FHIR (Wilkinson et al., 2016; Vorisek et al., 2022). When raw data cannot be accessed, variable definitions and processing metadata should at least be retained, and bridging validation should be conducted when devices or algorithms are changed (Woolley et al., 2019).

Accompanying the above issues of storage and platform dependence are more complex privacy risks (Canali et al., 2022; Capulli et al., 2025). Uploading wearable and multi-omics data to cloud services or third-party platforms may facilitate centralized storage, model training, and cross-source integration, but it also expands the scope of access, attack surfaces, and boundaries of responsibility, while potentially raising concerns regarding data ownership, secondary use, and platform migration (Capulli et al., 2025; Doherty et al., 2025). In particular, when continuous phenotypic data, omics data, geolocation information, and health records are linked, long-term physiological sequences may form identifiable “digital fingerprints” even after direct identifiers have been removed, and conventional de-identification may not completely prevent individual re-identification (Chikwetu et al., 2023). Therefore, WMRE2030 should follow the principle of “privacy by design, “ collect only the data necessary for decision-making, specify their purposes, retention periods, and access permissions, and implement whole-process governance through data segregation, encryption, tiered authorization, access auditing, and revocable consent (Capulli et al., 2025; Doherty et al., 2025). Edge computing, federated learning, and differential privacy may reduce the centralized transmission of raw data, but they can only mitigate rather than eliminate the risk of data leakage; contextual trade-offs are still required among model performance, computational burden, and regulatory requirements (Crowson et al., 2022; Chikwetu et al., 2023).

### 8.2 Algorithmic transparency and safety brakes

Within WMRE2030, artificial intelligence may directly participate in generating exercise recommendations. The consequences of error would therefore not be limited to predictive bias, but could further translate into inappropriate exercise exposure and even potential health and safety risks (Lekadir et al., 2024). Against this background, algorithmic transparency becomes a key foundation for ensuring trustworthy system operation (Liu et al., 2020). Transparency here does not mean that complete source code must be disclosed to all users; rather, it requires the system to provide adequate traceability and clearly explain the data and data quality underlying each recommendation, the populations and contexts to which the model applies, the current level of predictive uncertainty, the conditions that have exceeded the validated scope of the model, and whether the final decision was made by the algorithm or by a professional (Liu et al., 2020; Food and Administration, 2024; Lekadir et al., 2024). Relevant documents from FUTURE-AI and the World Health Organization likewise emphasize that trustworthy health artificial intelligence should comprehensively satisfy requirements relating to traceability, robustness, explainability, data quality assurance, risk management, and human oversight (Organization, 2023; Lekadir et al., 2024).

In addition, the establishment of “safety brakes” should not remain at the level of abstract principles. WMRE2030 could operationalize them in the future as a tiered and progressive fail-safe protocol (Guidance, 2021). The first level is data-integrity gating: when signal loss, obvious motion artifacts, timestamp misalignment, device malfunction, or physiologically implausible data are detected, model updating should be suspended immediately to avoid generating new exercise prescriptions from low-quality inputs. The second level is model-applicability gating: when inputs exceed the system’s validated scope of application, deviate substantially from the model’s training distribution, show unexplained conflicts across information sources, or produce uncertainty above a predefined threshold, the system should reduce the level of automation and either maintain the current prescription or revert to a pre-specified conservative plan grounded in established evidence, such as the ACSM’s Guidelines for Exercise Testing and Prescription (Ozemek et al., 2025), the WHO Guidelines on Physical Activity and Sedentary Behaviour (Organization, 2020), and relevant population-specific clinical exercise guidelines. The third level is physiological-risk gating: when a single indicator exceeds a safety threshold, multiple indicators display persistent adverse trends, or risk signals are repeatedly triggered, the system should prioritize reducing exercise load, shortening the validity period of the prescription, or postponing exercise rather than continuing to optimize individualized performance. The fourth level is human-escalation gating: when uncertainty persists, multiple risk signals cluster, or clinical warning signs emerge, automated prescription updating should be suspended and human review initiated according to the level of risk, with referral, when necessary, to a physician, exercise physiologist, coach, or other relevant professional for further assessment. These safeguards should always take precedence over performance-optimization objectives. Their triggering conditions, fallback rules, and escalation pathways should be prospectively defined before system deployment, while input status, model version, trigger rationale, prescription adjustment, and human intervention should be fully documented to ensure that the entire process is auditable and traceable and can be calibrated according to the risk profiles of different populations.

### 8.3 Cost, accessibility, and collaborative governance

From a broader technological perspective, one of the core values of artificial intelligence and automation is to transfer repetitive, standardized, and data-intensive tasks from humans to intelligent systems, thereby reducing labor and service costs while providing continuous and individualized support at a larger scale (Wenderott et al., 2024; El Arab and Al Moosa, 2025). WMRE2030 follows this logic by attempting to reduce sustained dependence on labor-intensive human assessment and progressively lower the marginal cost of precision exercise services. However, during the early transition from a conceptual framework to real-world application, its development, validation, and iteration would still require substantial investment, including multi-source data collection, omics testing, device maintenance, algorithm training, cloud computing, professional review, and continuous trials in clinical, community, and competitive sports settings (Greenhalgh et al., 2017; Vasey et al., 2022; Lekadir et al., 2024; El Arab and Al Moosa, 2025). More importantly, even after repeated optimization has produced a stable system, it will still be necessary to determine whether real-world accessibility expands in parallel with reductions in cost (Wilson et al., 2024; Organization, 2025). If declining costs do not translate into broader service accessibility, the benefits of WMRE2030 may remain concentrated among resource-rich institutions and individuals with greater ability to pay, whereas less-developed regions, where physical inactivity and chronic disease burdens are often more pronounced, may continue to benefit less (Canali et al., 2022; Organization, 2022b, Organization, 2022a; Wilson et al., 2024). Technological progress could therefore further reinforce existing disparities in health resources, creating a global precision-exercise equity paradox characterized by a “Matthew effect” (Canali et al., 2022; Wilson et al., 2024). Future studies should therefore evaluate not only whether WMRE2030 is “effective, “ but also its cost-effectiveness, resource requirements, scalability, and equity implications, while developing tiered implementation strategies ranging from a minimum sufficient configuration to high-resource configurations for different settings (Wilson et al., 2024; El Arab and Al Moosa, 2025; Organization, 2025). Only when technological advances are genuinely translated into lower service costs, broader coverage, and more equitable access can WMRE2030 evolve from a high-cost precision prototype into sustainable infrastructure serving “fitness for all” (Wilson et al., 2024; Organization, 2025).

It should also be emphasized that the implementation and translation of WMRE2030 cannot be accomplished by the field of exercise science alone. Researchers in exercise science, clinical medicine, bioinformatics, artificial intelligence, and public health will need to jointly establish the evidence base, while wearable-device companies, omics-testing institutions, data platforms, and software developers will need to provide interoperable technical conditions. At the same time, legal and ethics experts, regulatory agencies, government departments, and policy makers will need to jointly clarify data ownership, privacy protection, boundaries of responsibility, access standards, and payment mechanisms (Greenhalgh et al., 2017; Lekadir et al., 2024; Organization, 2025). Community organizations, healthcare institutions, sports organizations, and end users should also participate in needs definition and implementation evaluation to ensure that the system is not merely technically feasible, but can be genuinely embedded within the real-world workflows of different regions and populations (Lekadir et al., 2024; Wilson et al., 2024). Accordingly, the real-world deployment of WMRE2030 should be regarded as a collaborative governance process involving research, industry, policy, law, and society, rather than as a technology-transfer process driven by a single discipline (Greenhalgh et al., 2017; Lekadir et al., 2024; Organization, 2025) (**Figure 4**).

**Figure 3:**
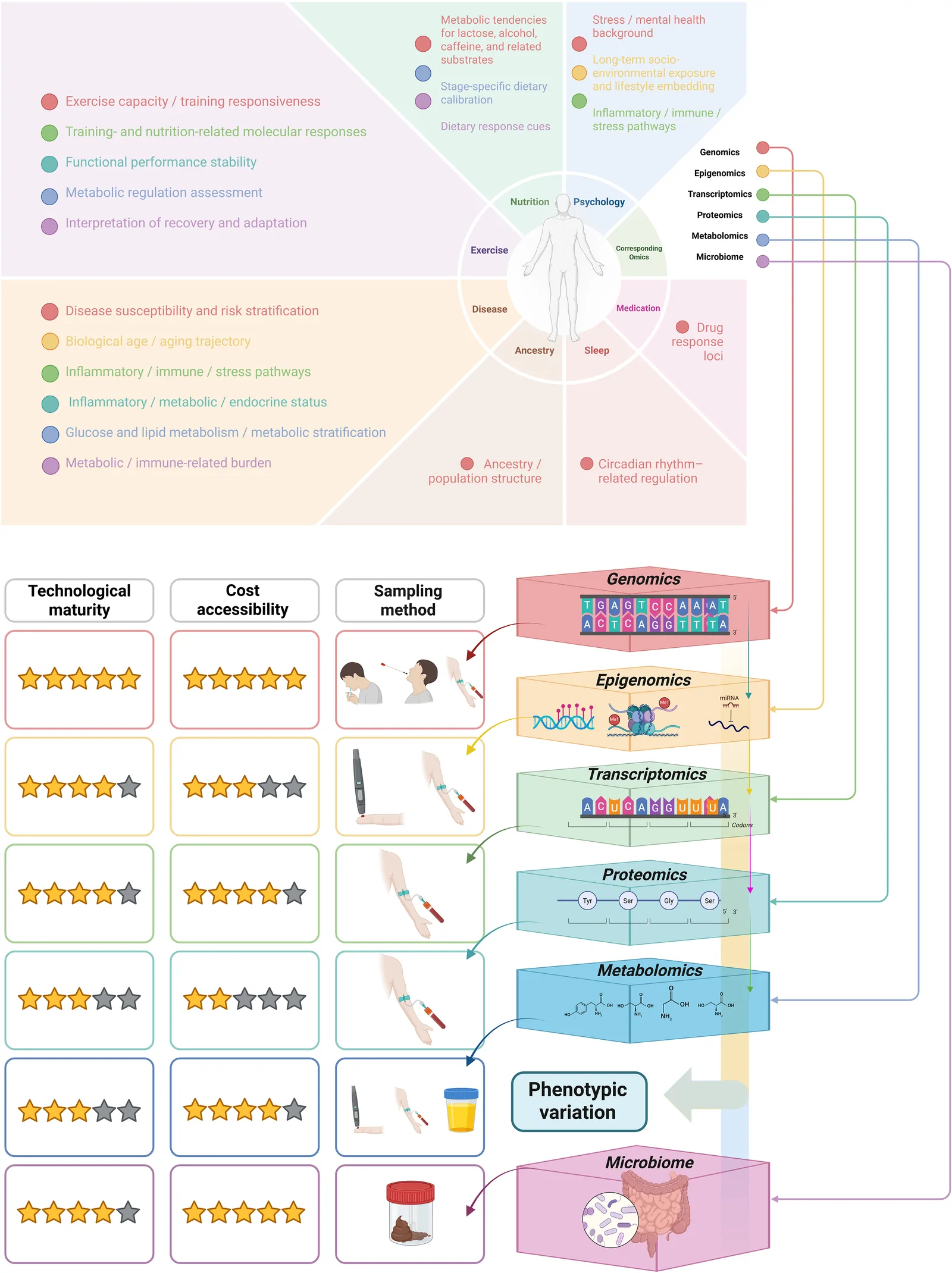
Real-world application space and accessibility of multi-omics in precision exercise and health. The upper half of the circle represents the actual multi-omics applications provided in the current real-world market and research service systems, rather than theoretical potential applications. The star ratings were derived using a predefined, evidence-informed, semi-quantitative ordinal framework. Technological maturity was evaluated according to platform and protocol standardization, quality-control and analytical workflow maturity, reproducibility and cross-platform comparability, scale of research or translational use, and availability beyond specialized laboratories. Cost accessibility was evaluated by considering representative per-sample prices, sampling and preprocessing burden, infrastructure requirements, batch scalability, and availability through commercial or institutional core facilities. Ratings range from one star, indicating very limited relative readiness or accessibility, to five stars, indicating very high relative readiness or accessibility. These ratings are intended as comparative implementation-readiness categories rather than a validated quantitative index. Where available evidence was heterogeneous, conservative author judgement was applied. Full operational definitions, layer-specific rationales, representative cost ranges, evidence sources, URLs, and access dates are provided in **Supplementary Table 2**.

**Figure 4:**
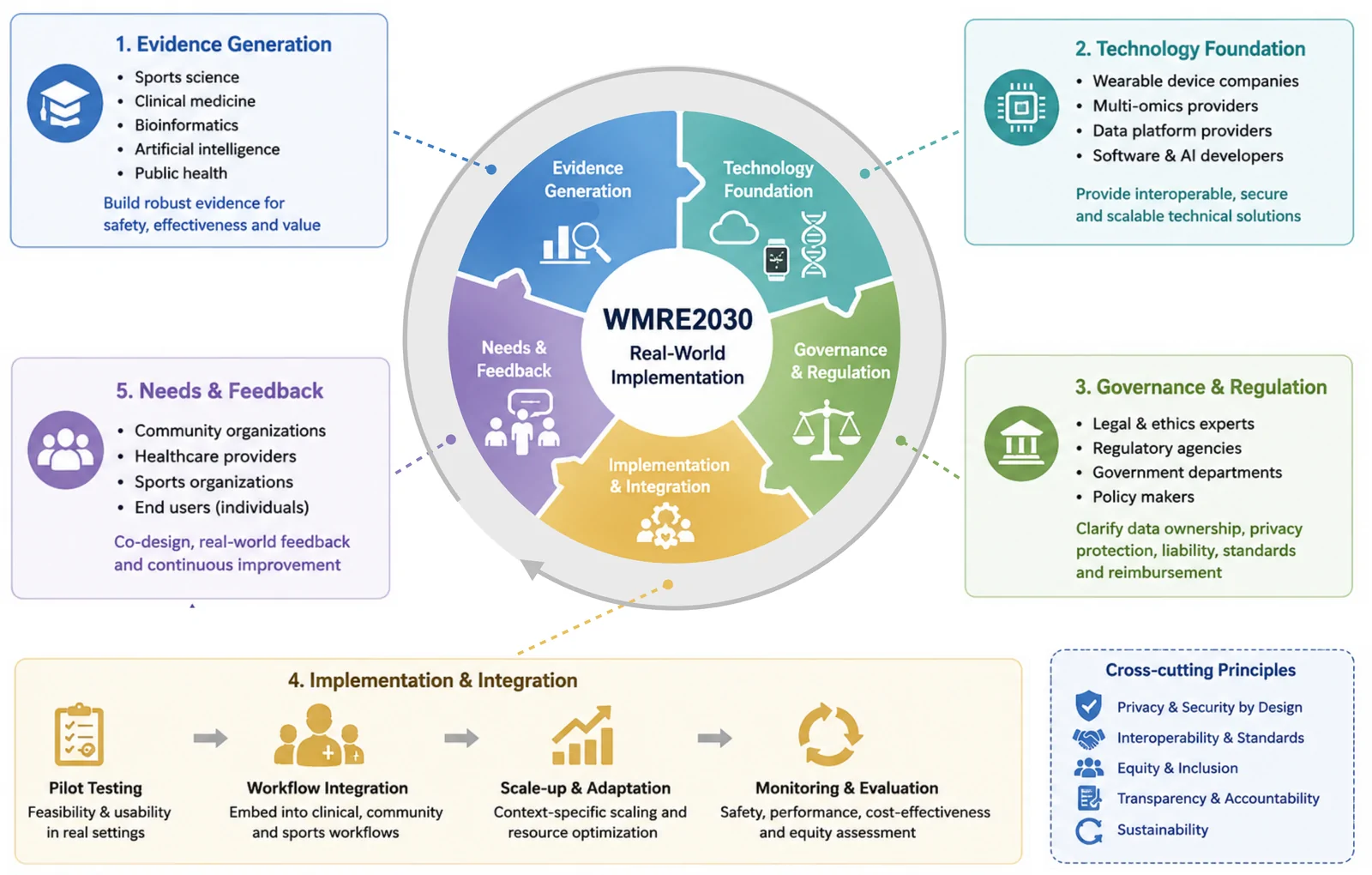
Multi-stakeholder implementation and governance framework for the real-world deployment of WMRE2030. AI, artificial intelligence; WMRE2030, *Wearables* Multi-Omics, AI-Driven Real-Time Feedback, and Exercise 2030.

### 8.4 Minimum-viable WMRE2030 large language model

As large language models increasingly participate in the retrieval, organization, and utilization of scientific knowledge, future academic papers may no longer serve only as static knowledge carriers for human readers, but may also become structured inputs through which artificial intelligence understands specific theoretical frameworks, rule systems, and decision processes (Lewis et al., 2020; Asai et al., 2026). Based on this possible direction of development, WMRE2030 could also explore extending from a conceptual methodological framework toward an operational specification that can be read by both humans and machines (Lewis et al., 2020; Asai et al., 2026). Specifically, readers may attempt to input this paper into a general-purpose or locally deployed large language model and combine it with the standardized prompt instructions provided in the supplementary materials (**Supplementary Table 3** and **4**), thereby guiding the model to organize information according to the day-scale closed-loop logic of “state sensing-constraint interpretation-decision support-exercise execution-response feedback” (Lewis et al., 2020), as shown in **Figure 5**. The resulting system may be regarded as a “minimum-viable WMRE2030 prototype” intended for interactive reader experience. It should be emphasized that this prototype is designed only for conceptual experience and methodological exploration and is not equivalent to a precision-exercise domain model that has undergone professional training, external validation, or clinical evaluation (Lekadir et al., 2024).

**Figure 5:**
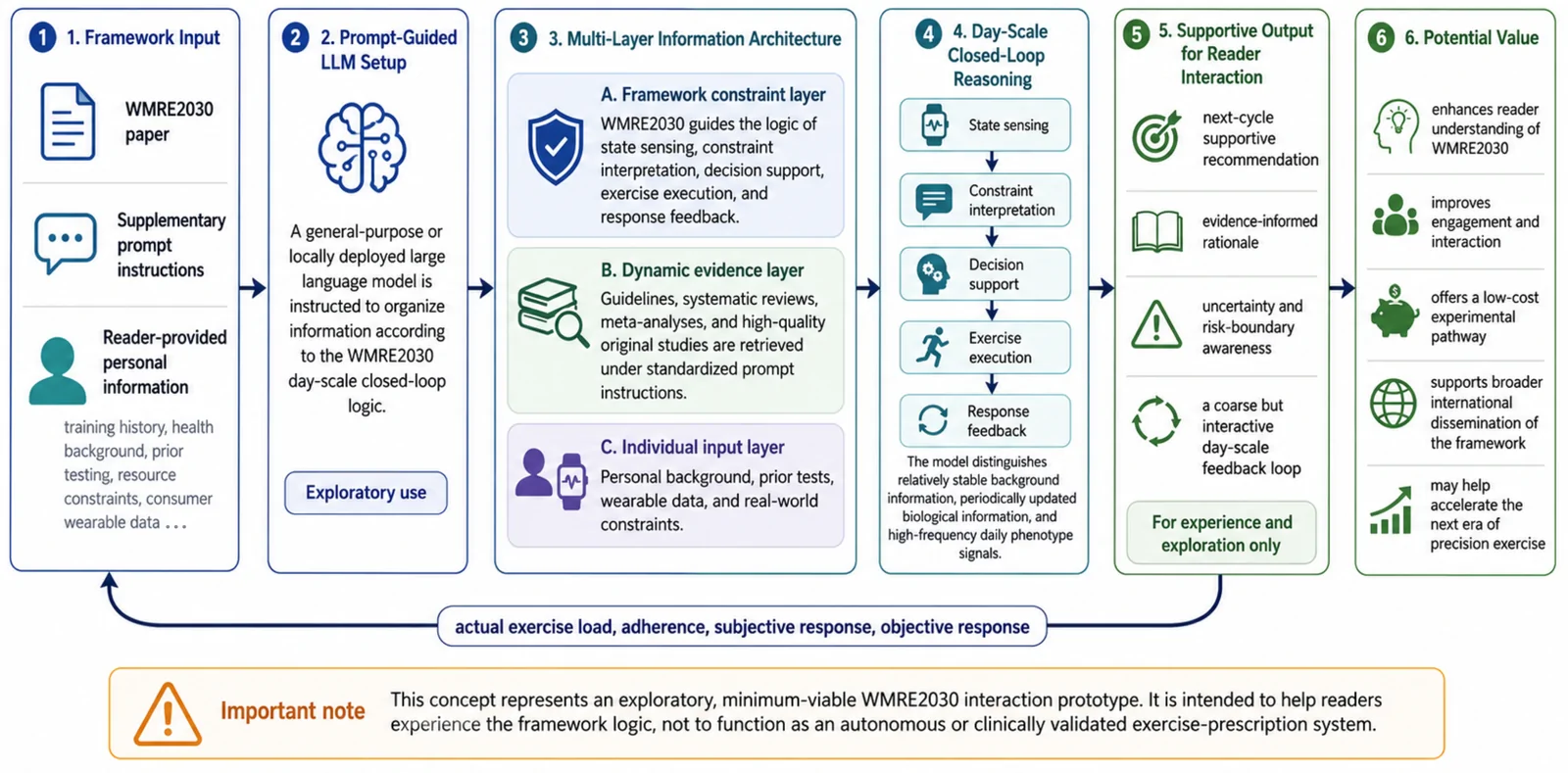
Interactive workflow of a minimum-viable WMRE2030 large language model prototype. LLM, large language model; *WMRE2030*, Wearables, Multi-Omics, AI-Driven Real-Time Feedback, and Exercise 2030.

In this exploratory configuration, the WMRE2030 paper could serve as a framework-constraint layer for the model’s understanding and organization of tasks. Professional guidelines, systematic reviews, meta-analyses, and high-quality original studies automatically screened under the requirements of standardized prompt instructions could form a dynamic evidence layer (Lewis et al., 2020; Zhang et al., 2025; Asai et al., 2026), while previous personal tests, health and training background, real-world resource conditions, and consumer-grade wearable data could be used experimentally as an individual input layer (Fang et al., 2024; Khasentino et al., 2025). The model could be guided to distinguish relatively stable background information, periodically updated biological information, and high-frequency daily phenotypes, and, under the constraints of the prompt protocol, to preliminarily assess data quality, evidence applicability, uncertainty, and risk boundaries before generating supportive information for the next decision cycle (Lekadir et al., 2024; Khasentino et al., 2025). The actual load, adherence, subjective perceptions, and objective responses after exercise could then be re-entered into the model to simulate a day-scale feedback loop that is relatively coarse-grained but has basic interactive characteristics (Fang et al., 2024; Khasentino et al., 2025). This interactive experience may, to some extent, improve readers’ understanding of and engagement with the operational logic of WMRE2030, while providing a relatively low-cost experimental pathway for examining the framework’s operability and facilitating its international dissemination, with the aim of helping to accelerate global precision exercise into a new era.

## 9 Summary and outlook

This review proposes WMRE2030 as a day-scale methodological framework for precision exercise, integrating wearable sensing, multi-omics constraints, AI-supported decision organization, and exercise execution within a continuously updating feedback loop. Its contribution lies not in assembling emerging technologies, but in assigning them distinct and complementary roles within a common architecture: wearables characterize dynamic states, multi-omics provides biological context and applicability boundaries, AI organizes evidence-constrained decisions, and exercise supplies the actionable output through which the system learns. The principle of “the same architecture, different parameters” further offers a potentially transferable pathway across clinical populations, the general public, and high-performance athletes. Nevertheless, WMRE2030 should be regarded as a testable methodological roadmap rather than a validated prescriptive system. Future research should progressively examine its incremental value over conventional exercise prescription through longitudinal, micro-randomized, and multicentre studies; establish interoperable data standards and externally validated decision rules; and prospectively define uncertainty thresholds, safety brakes, and human-escalation pathways. Equally important, evaluation should extend beyond predictive accuracy or physiological efficacy to include cost-effectiveness, usability, privacy, scalability, and equitable access. Minimum-viable implementations may provide a pragmatic starting point, but increasing automation should occur only in proportion to accumulated evidence and demonstrated safety. Ultimately, the significance of WMRE2030 will depend not on whether it can generate ever more individualized recommendations, but on whether it can convert heterogeneous personal data into decisions that are biologically interpretable, auditable, clinically or practically meaningful, and sustainably embedded in real-world exercise systems.

## References

[B1] Albers D. Sirlanci M. Levine M. Claassen J. Der Nigoghossian C. Hripcsak G. (2023). Interpretable physiological forecasting in the ICU using constrained data assimilation and electronic health record data. J. Biomed. Inf. 145, 104477. doi: 10.1016/j.jbi.2023.104477 37604272

[B2] Alfonso C. Garcia-Gonzalez M. A. Parrado E. Gil-Rojas J. Ramos-Castro J. Capdevila L. (2022). Agreement between two photoplethysmography-based wearable devices for monitoring heart rate during different physical activity situations: a new analysis methodology. Sci. Rep. 12, 15448. doi: 10.1038/s41598-022-18356-9 36104356 PMC9474518

[B3] Allen J. (2007). Photoplethysmography and its application in clinical physiological measurement. Physiol. Meas. 28, R1–R39. doi: 10.1088/0967-3334/28/3/r01 17322588

[B4] Angelucci A. Cavicchioli M. Cintorrino I. A. Lauricella G. Rossi C. Strati S. . (2021). Smart textiles and sensorized garments for physiological monitoring: a review of available solutions and techniques. Sens. 21, 814. doi: 10.3390/s21030814 33530403 PMC7865961

[B5] Apsley A. T. Ye Q. Caspi A. Chiaro C. Etzel L. Hastings W. J. . (2025). Cross‐tissue comparison of epigenetic aging clocks in humans. Aging Cell 24, e14451. doi: 10.1111/acel.14451 39780748 PMC11984668

[B6] Arquilla K. Webb A. K. Anderson A. P. (2020). Textile electrocardiogram (ECG) electrodes for wearable health monitoring. Sensors 20, 1013. doi: 10.3390/s20041013 32069937 PMC7070603

[B7] Asai A. He J. Shao R. Shi W. Singh A. Chang J. C. . (2026). Synthesizing scientific literature with retrieval-augmented language models. Nature 650, 1–7. doi: 10.1038/s41586-025-10072-4 41639446 PMC12935541

[B8] Ates H. C. Nguyen P. Q. Gonzalez-Macia L. Morales-Narváez E. Güder F. Collins J. J. . (2022). End-to-end design of wearable sensors. Nat. Rev. Mater. 7, 887–907. doi: 10.1038/s41578-022-00460-x 35910814 PMC9306444

[B9] Babu M. Snyder M. (2023). Multi-omics profiling for health. Mol. Cell. Proteomics 22, 100561. doi: 10.1016/j.mcpro.2023.100561 37119971 PMC10220275

[B10] Baccarelli A. Dolinoy D. C. Walker C. L. (2023). A precision environmental health approach to prevention of human disease. Nat. Commun. 14, 2449. doi: 10.1038/s41467-023-37626-2 37117186 PMC10147599

[B11] Baltrušaitis T. Ahuja C. Morency L.-P. (2018). Multimodal machine learning: a survey and taxonomy. IEEE Trans. Pattern Anal. Mach. Intell. 41, 423–443. doi: 10.1109/TPAMI.2018.2798607 29994351

[B12] Beato M. Coratella G. Stiff A. Iacono A. D. (2018). The validity and between-unit variability of GNSS units (STATSports Apex 10 and 18 Hz) for measuring distance and peak speed in team sports. Front. Physiol. 9, 1288. doi: 10.3389/fphys.2018.01288 30298015 PMC6161633

[B13] Bell C. G. Beck S. (2010). The epigenomic interface between genome and environment in common complex diseases. Briefings Funct. Genomics 9, 477–485. doi: 10.1093/bfgp/elq026 21062751 PMC3080746

[B14] Bourdon P. C. Cardinale M. Murray A. Gastin P. Kellmann M. Varley M. C. . (2017). Monitoring athlete training loads: consensus statement. Int. J. Sports Physiol. Perform. 12, S2-161–S2-170. doi: 10.1123/IJSPP.2017-0208 28463642

[B15] Breteler M. J. Huizinga E. van Loon K. Leenen L. P. Dohmen D. A. Kalkman C. J. . (2018). Reliability of wireless monitoring using a wearable patch sensor in high-risk surgical patients at a step-down unit in the Netherlands: a clinical validation study. BMJ Open 8, e020162. doi: 10.1136/bmjopen-2017-020162 29487076 PMC5855309

[B16] Buford T. W. Roberts M. D. Church T. S. (2013). Toward exercise as personalized medicine. Sports Med. 43, 157–165. doi: 10.1007/s40279-013-0018-0 23382011 PMC3595541

[B17] Bujang N. A. Ghosh C. Palaniappan K. Vogel S. Lim J. C. Tiong W. W. . (2025). Non-clinical direct-to-consumer genetic testing: a scoping review of regulatory frameworks and issues. Health Policy Technol. 14, 101106. doi: 10.1016/j.hlpt.2025.10110642574925

[B18] Bullock G. S. Mylott J. Hughes T. Nicholson K. F. Riley R. D. Collins G. S. (2022). Just how confident can we be in predicting sports injuries? A systematic review of the methodological conduct and performance of existing musculoskeletal injury prediction models in sport. Sports Med. 52, 2469–2482. doi: 10.1007/s40279-022-01698-9 35689749

[B19] Canali S. Schiaffonati V. Aliverti A. (2022). Challenges and recommendations for wearable devices in digital health: data quality, interoperability, health equity, fairness. PloS Digital Health 1, e0000104. doi: 10.1371/journal.pdig.0000104 36812619 PMC9931360

[B20] Cao R. Azimi I. Sarhaddi F. Niela-Vilen H. Axelin A. Liljeberg P. . (2022). Accuracy assessment of oura ring nocturnal heart rate and heart rate variability in comparison with electrocardiography in time and frequency domains: comprehensive analysis. J. Med. Internet Res. 24, e27487. doi: 10.2196/27487 35040799 PMC8808342

[B21] Capulli E. Druda Y. Palmese F. Butt A. H. Domenicali M. Macchiarelli A. G. . (2025). Ethical and legal implications of health monitoring wearable devices: a scoping review. Soc Sci. Med. 370, 117685. doi: 10.1016/j.socscimed.2025.117685 40010231

[B22] Chen R. Mias G. I. Li-Pook-Than J. Jiang L. Lam H. Y. Chen R. . (2012). Personal omics profiling reveals dynamic molecular and medical phenotypes. Cell. 148, 1293–1307. doi: 10.1016/j.cell.2012.02.009 22424236 PMC3341616

[B23] Chikwetu L. Miao Y. Woldetensae M. K. Bell D. Goldenholz D. M. Dunn J. (2023). Does deidentification of data from wearable devices give us a false sense of security? A systematic review. Lancet Digit. Health 5, e239–e247. doi: 10.1016/s2589-7500(22)00234-5 36797124 PMC10040444

[B24] Choi W. Kim S.-H. Lee W. Kang S.-H. Yoon C.-H. Youn T.-J. . (2020). Comparison of continuous ECG monitoring by wearable patch device and conventional telemonitoring device. J. Korean Med. Sci. 35, e363. doi: 10.3346/jkms.2020.35.e363 33200590 PMC7669461

[B25] Cosoli G. Spinsante S. Scalise L. (2020). Wrist-worn and chest-strap wearable devices: systematic review on accuracy and metrological characteristics. Measurement 159, 107789. doi: 10.1016/j.measurement.2020.10778942574925

[B26] Cowan R. E. (2016). Exercise is medicine initiative: physical activity as a vital sign and prescription in adult rehabilitation practice. Arch. Phys. Med. Rehabil. 97, S232–S237. doi: 10.1016/j.apmr.2016.01.040 27470321

[B27] Cramer L. A. Wimmer M. A. Malloy P. O’Keefe J. A. Knowlton C. B. Ferrigno C. (2022). Validity and reliability of the Insole3 instrumented shoe insole for ground reaction force measurement during walking and running. Sens. 22, 2203. doi: 10.3390/s22062203 35336374 PMC8951440

[B28] Cross J. L. Choma M. A. Onofrey J. A. (2024). Bias in medical AI: implications for clinical decision-making. PloS Digital Health 3, e0000651. doi: 10.1371/journal.pdig.0000651 39509461 PMC11542778

[B29] Crowson M. G. Moukheiber D. Arévalo A. R. Lam B. D. Mantena S. Rana A. . (2022). A systematic review of federated learning applications for biomedical data. PloS Digital Health 1, e0000033. doi: 10.1371/journal.pdig.0000033 36812504 PMC9931322

[B30] Cui M. Deng F. Disis M. L. Cheng C. Zhang L. (2024). Advances in the clinical application of high-throughput proteomics. Explor. Res. hypothesis Med. 9, 209. doi: 10.14218/erhm.2024.00006 39148720 PMC11326426

[B31] Dagher M. Ongo G. Robichaud N. Kong J. Rho W. Teahulos I. . (2025). nELISA: A high-throughput, high-plex platform enables quantitative profiling of the inflammatory secretome. Nat. Methods 22, 1–11. doi: 10.1038/s41592-025-02861-6 41203862 PMC12615254

[B32] Daniore P. Nittas V. Haag C. Bernard J. Gonzenbach R. von Wyl V. (2024). From wearable sensor data to digital biomarker development: ten lessons learned and a framework proposal. NPJ Digital Med. 7, 161. doi: 10.1038/s41746-024-01151-3 38890529 PMC11189504

[B33] Dawson L. McErlain-Naylor S. A. Devereux G. Beato M. (2026). Interunit reliability of STATSports APEX global navigation satellite system and accelerometer-derived metrics during shuttle run protocols of varied distances and change of direction frequency. J. Sports Sci. 44, 117–129. doi: 10.1080/02640414.2025.2555554 40931543

[B34] Delves R. I. M. Aughey R. J. Ball K. Duthie G. M. (2021). The quantification of acceleration events in elite team sport: a systematic review. Sports Med. - Open 7, 45. doi: 10.1186/s40798-021-00332-8 34191142 PMC8245618

[B35] De Zambotti M. Rosas L. Colrain I. M. Baker F. C. (2019). The sleep of the ring: comparison of the ŌURA sleep tracker against polysomnography. Behav. Sleep Med. 17, 124–136. doi: 10.1080/15402002.2017.1300587 28323455 PMC6095823

[B36] Dezem F. S. Arjumand W. DuBose H. Morosini N. S. Plummer J. (2024). Spatially resolved single-cell omics: methods, challenges, and future perspectives. Annu. Rev. Biomed. Data Sci. 7, 131–153. doi: 10.1146/annurev-biodatasci-102523-103640 38768396

[B37] Diabetes Care (2025). 7. diabetes technology: standards of care in diabetes—2025. Diabetes Care 48, S146–S166. doi: 10.2337/dc25-S007 39651978 PMC11635043

[B38] Dial M. B. Hollander M. E. Vatne E. A. Emerson A. M. Edwards N. A. Hagen J. A. (2025). Contextual equivalence for accurate comparison of wearables requires transparency. Physiol. Rep. 13, e70706. doi: 10.14814/phy2.70706 41399178 PMC12706116

[B39] Ding H. Ho K. Searls E. Low S. Li Z. Rahman S. . (2024). Assessment of wearable device adherence for monitoring physical activity in older adults: pilot cohort study. JMIR Aging 7, e60209. doi: 10.2196/60209 39454101 PMC11530080

[B40] Doherty C. Baldwin M. Lambe R. Altini M. Caulfield B. (2025). Privacy in consumer wearable technologies: a living systematic analysis of data policies across leading manufacturers. NPJ Digital Med. 8, 363. doi: 10.1038/s41746-025-01757-1 40517175 PMC12167361

[B41] Downey C. Ng S. Jayne D. Wong D. (2019). Reliability of a wearable wireless patch for continuous remote monitoring of vital signs in patients recovering from major surgery: a clinical validation study from the TRaCINg trial. BMJ Open 9, e031150. doi: 10.1136/bmjopen-2019-031150 31420399 PMC6701670

[B42] Düking P. Robertson S. Holmberg H.-C. Wolf K.-H. Sperlich B. (2025). Classification system for AI-enabled consumer-grade wearable technologies aiming to automatize decision-making about individualization of exercise procedures. Front. Sports Active Living 6, 1500563. doi: 10.3389/fspor.2024.1500563 PMC11813931 39945004

[B43] Dunn J. Kidzinski L. Runge R. Witt D. Hicks J. L. Rose S. M. S-F. . (2021). Wearable sensors enable personalized predictions of clinical laboratory measurements. Nat. Med. 27, 1105–1112. doi: 10.1038/s41591-021-01339-0 34031607 PMC8293303

[B44] El Arab R. A. Al Moosa O. A. (2025). Systematic review of cost effectiveness and budget impact of artificial intelligence in healthcare. NPJ Digital Med. 8, 548. doi: 10.1038/s41746-025-01722-y 40858882 PMC12381244

[B45] Elstub L. J. Nurse C. A. Grohowski L. M. Volgyesi P. Wolf D. N. Zelik K. E. (2022). Tibial bone forces can be monitored using shoe-worn wearable sensors during running. J. Sports Sci. 40, 1741–1749. doi: 10.1080/02640414.2022.2107816 35938189 PMC9938946

[B46] Fang C. M. Danry V. Whitmore N. Bao A. Hutchison A. Pierce C. . (2024). “ Physiollm: supporting personalized health insights with wearables and large language models”, in: 2024 IEEE EMBS International Conference on Biomedical and Health Informatics (BHI) (Piscataway, NJ, United State: IEEE), 1–8. doi: 10.1109/BHI62660.2024.10913781

[B47] Faust L. Cui J. Knepper C. Nasseri M. Worrell G. Brinkmann B. H. (2025). Detecting diverse seizure types with wrist-worn wearable devices: a comparison of machine learning approaches. Sens. 25, 5562. doi: 10.3390/s25175562 40942991 PMC12431448

[B48] FDA, U. S (2018). De novo classification request for irregular rhythm notification feature. DEN180042.

[B49] Ferraz A. Duarte-Mendes P. Sarmento H. Valente-Dos-Santos J. Travassos B. (2023). Tracking devices and physical performance analysis in team sports: a comprehensive framework for research—trends and future directions. Front. Sports Active Living 5, 1284086. doi: 10.3389/fspor.2023.1284086 38077284 PMC10708914

[B50] Food and Administration (2024). “ Transparency for machine learning-enabled medical devices: guiding principles,” in Us Food Drug Adm. Retrieved June, Silver Spring, MD, United States: U.S. Food and Drug Administration (FDA). 30.

[B51] Frodi D. M. Kolk M. Z. Langford J. Knops R. Tan H. L. Andersen T. O. . (2024). Long-term adherence to a wearable for continuous behavioural activity measuring in the SafeHeart implantable cardioverter defibrillator population. Eur. Heart J. Digital Health 5, 622–632. doi: 10.1093/ehjdh/ztae055 39318686 PMC11417489

[B52] Fuller D. Colwell E. Low J. Orychock K. Tobin M. A. Simango B. . (2020b). Reliability and validity of commercially available wearable devices for measuring steps, energy expenditure, and heart rate: systematic review. JMIR mHealth uHealth 8, e18694. doi: 10.2196/18694 32897239 PMC7509623

[B53] Fuller A. Fan Z. Day C. Barlow C. (2020a). Digital twin: Enabling technologies, challenges and open research. IEEE Access 8, 108952–108971. doi: 10.1109/ACCESS.2020.299835825079929

[B54] Gao P. Shen X. Zhang X. Jiang C. Zhang S. Zhou X. . (2022). Precision environmental health monitoring by longitudinal exposome and multi-omics profiling. Genome Res. 32, 1199–1214. doi: 10.1101/gr.276521.121 35667843 PMC9248886

[B55] Gazi A. H. Gao D. Ghosh S. Xu Z. Trella A. L. Klasnja P. . (2026). Digital twins for just-in-time adaptive interventions (jitais): Framework for optimizing and continually improving jitais. J. Med. Internet Res. 28, e72830. doi: 10.2196/72830 41813230 PMC12978917

[B56] Ghassemi M. Oakden-Rayner L. Beam A. L. (2021). The false hope of current approaches to explainable artificial intelligence in health care. Lancet digit. Health 3, e745–e750. doi: 10.1016/S2589-7500(21)00208-9 34711379

[B57] Gil E. Orini M. Bailon R. Vergara J. M. Mainardi L. Laguna P. (2010). Photoplethysmography pulse rate variability as a surrogate measurement of heart rate variability during non-stationary conditions. Physiol. Meas. 31, 1271–1290. doi: 10.1088/0967-3334/31/9/015 20702919

[B58] Golbus J. R. Dempsey W. Jackson E. A. Nallamothu B. K. Klasnja P. (2021). Microrandomized trial design for evaluating just-in-time adaptive interventions through mobile health technologies for cardiovascular disease. Circ. Cardiovasc. Qual. Outcomes 14, e006760. doi: 10.1161/CIRCOUTCOMES.120.006760 33430608 PMC8887814

[B59] Gong E. J. Bang C. S. Lee J. J. Baik G. H. (2025). Smart ring in clinical medicine: A systematic review. Biomimetics 10, 819. doi: 10.3390/biomimetics10120819 41439888 PMC12730986

[B60] Greenhalgh T. Wherton J. Papoutsi C. Lynch J. Hughes G. Hinder S. . (2017). Beyond adoption: a new framework for theorizing and evaluating nonadoption, abandonment, and challenges to the scale-up, spread, and sustainability of health and care technologies. J. Med. Internet Res. 19, e8775. doi: 10.2196/jmir.8775 29092808 PMC5688245

[B61] Gu F. Meyer A. J. Ježek F. Zhang S. Catalan T. Miller A. . (2025). Identification of digital twins to guide interpretable AI for diagnosis and prognosis in heart failure. NPJ Digital Med. 8, 110. doi: 10.1038/s41746-025-01501-9 39966509 PMC11836287

[B62] Guasch-Ferré M. Wittenbecher C. Palmnäs M. Ben-Yacov O. Blaak E. E. Dahm C. C. . (2025). Precision nutrition for cardiometabolic diseases. Nat. Med. 31, 1–10. doi: 10.1038/s41591-025-03669-9 40307513

[B63] Guidance W. H. O. (2021). Ethics and governance of artificial intelligence for health. World Health Organ., 1–165.

[B64] Hajjar G. Santos M. C. B. Bertrand-Michel J. Canlet C. Castelli F. Creusot N. . (2023). Scaling-up metabolomics: Current state and perspectives. TrAC Trends Anal. Chem. 167, 117225. doi: 10.1016/j.trac.2023.11722542574925

[B65] Hauskeller M. Shore L. (2025). What are the most ethically salient implications of epigenetic age testing? AMA J. Ethics 27, 828–833. doi: 10.1001/amajethics.2025.828 41411396

[B66] Hliš T. Fister I. Fister I. Jr (2024). Digital twins in sport: Concepts, taxonomies, challenges and practical potentials. Expert Syst. Appl. 258, 125104. doi: 10.1016/j.eswa.2024.12510442574925

[B67] Hoffmann D. E. Von Rosenvinge E. C. Roghmann M.-C. Palumbo F. B. McDonald D. Ravel J. (2024). The DTC microbiome testing industry needs more regulation. Science 383, 1176–1179. doi: 10.1126/science.adk4271 38484067 PMC13387485

[B68] Hood L. Flores M. (2012). A personal view on systems medicine and the emergence of proactive P4 medicine: predictive, preventive, personalized and participatory. New Biotechnol. 29, 613–624. doi: 10.1016/j.nbt.2012.03.004 22450380

[B69] Hoog Antink C. Mai Y. Peltokangas M. Leonhardt S. Oksala N. Vehkaoja A. (2021). Accuracy of heart rate variability estimated with reflective wrist-PPG in elderly vascular patients. Sci. Rep. 11, 8123. doi: 10.1038/s41598-021-87489-0 33854090 PMC8047026

[B70] Hubbard I. Beniczky S. Ryvlin P. (2021). The challenging path to developing a mobile health device for epilepsy: the current landscape and where we go from here. Front. Neurol. 12, 740743. doi: 10.3389/fneur.2021.740743 34659099 PMC8517120

[B71] Hull L. E. Aday A. W. Bui Q. M. Luzum J. A. Muchira J. M. Wand H. . (2025). Direct-to-consumer genetic testing for cardiovascular disease: A scientific statement from the american heart association. Circulation 151, e905–e917. doi: 10.1161/CIR.0000000000001304 40079117 PMC12093157

[B72] Hussain T. Ullah S. Fernández-García R. Gil I. (2023). Wearable sensors for respiration monitoring: A review. Sens. 23, 7518. doi: 10.3390/s23177518 37687977 PMC10490703

[B73] Jakicic J. M. Kohrt W. M. Houmard J. A. Miller M. E. Radom-Aizik S. MoTrPAC Study Group (2024). Molecular Transducers of Physical Activity Consortium (MoTrPAC): human studies design and protocol. J. Appl. Physiol. 137, 473–493. doi: 10.1152/japplphysiol.00102.2024 38634503 PMC11427038

[B74] Jandoo T. (2020). WHO guidance for digital health: what it means for researchers. DIGITAL Health 6, 2055207619898984. doi: 10.1177/2055207619898984 31949918 PMC6952850

[B75] Kairouz P. McMahan H. B. (2021). Advances and open problems in federated learning. Found. Trends Mach. Learn. 14, 1–210. doi: 10.1561/2200000083

[B76] Kane S. (2024). Leveraging digital health initiatives to enhance the effectiveness, equity and responsiveness of health systems. BMJ Glob. Health 9, e014626. doi: 10.1136/bmjgh-2023-014626 39097295 PMC11308869

[B77] Kantrowitz A. B. Ben-David K. Morris M. Wittels H. L. Wishon M. J. McDonald S. M. . (2025). Pulse rate variability is not the same as heart rate variability: findings from a large, diverse clinical population study. Front. Physiol. 16, 1630032. doi: 10.3389/fphys.2025.1630032 40809286 PMC12343505

[B78] Katsoulakis E. Wang Q. Wu H. Shahriyari L. Fletcher R. Liu J. . (2024). Digital twins for health: a scoping review. NPJ Digital Med. 7, 77. doi: 10.1038/s41746-024-01073-0 38519626 PMC10960047

[B79] Khasentino J. Belyaeva A. Liu X. Yang Z. Furlotte N. A. Lee C. . (2025). A personal health large language model for sleep and fitness coaching. Nat. Med. 31, 3394–3403. doi: 10.1038/s41591-025-03888-0 40813712 PMC12532688

[B80] Khoshdel (2010). The impact of arm position and pulse pressure on the validation of a wrist-cuff blood pressure measurement device in a high risk population. Int. J. Gen. Med. 3, 119. doi: 10.2147/IJGM.S7526 20463830 PMC2866552

[B81] Khoury M. J. Gwinn M. Glasgow R. E. Kramer B. S. (2012). A population perspective on how personalized medicine can improve health. Am. J. Prev. Med. 42, 639. doi: 10.1016/j.amepre.2012.02.012 PMC3629731 22608383

[B82] Kip H. Beerlage-de Jong N. van Gemert-Pijnen L. J. Kelders S. M. (2025). The CeHRes roadmap 2.0: update of a holistic framework for development, implementation, and evaluation of eHealth technologies. J. Med. Internet Res. 27, e59601. doi: 10.2196/59601 39805104 PMC11773290

[B83] Klasnja P. Hekler E. B. Shiffman S. Boruvka A. Almirall D. Tewari A. . (2015). Microrandomized trials: an experimental design for developing just-in-time adaptive interventions. Health Psychol. 34, 1220. doi: 10.1037/hea0000305 26651463 PMC4732571

[B84] Knoppers T. Beauchamp E. Dewar K. Kimmins S. Bourque G. Joly Y. . (2021). The omics of our lives: practices and policies of direct-to-consumer epigenetic and microbiomic testing companies. New Genet. Soc 40, 541–569. doi: 10.1080/14636778.2021.199757637339054

[B85] Köhler C. Bartschke A. Fürstenau D. Schaaf T. Salgado-Baez E. (2024). The value of smartwatches in the health care sector for monitoring, nudging, and predicting: Viewpoint on 25 years of research. J. Med. Internet Res. 26, e58936. doi: 10.2196/58936 39356287 PMC11549588

[B86] Kristiansson E. Fridolfsson J. Arvidsson D. Holmäng A. Börjesson M. Andersson-Hall U. (2023). Validation of Oura ring energy expenditure and steps in laboratory and free-living. BMC Med. Res. Methodol. 23, 50. doi: 10.1186/s12874-023-01868-x 36829120 PMC9950693

[B87] Kuwabara M. Harada K. Hishiki Y. Kario K. (2019). Validation of two watch‐type wearable blood pressure monitors according to the ANSI/AAMI/ISO81060‐2:2013 guidelines: Omron HEM‐6410T‐ZM and HEM‐6410T‐ZL. J. Clin. Hypertens. 21, 853–858. doi: 10.1111/jch.13499 30803128 PMC8030427

[B88] Laubenbacher R. Mehrad B. Shmulevich I. Trayanova N. (2024). Digital twins in medicine. Nat. Comput. Sci. 4, 184–191. doi: 10.1038/s43588-024-00607-6 38532133 PMC11102043

[B89] Leckey C. Van Dyk N. Doherty C. Lawlor A. Delahunt E. (2025). Machine learning approaches to injury risk prediction in sport: a scoping review with evidence synthesis. Br. J. Sports Med. 59, 491–500. doi: 10.1136/bjsports-2024-108576 39613453 PMC12013557

[B90] Lee S. Takano K. Yukita W. Tagawa Y. Sun L. Wang S. . (2025). A body-scale textile-based electromyogram monitoring system with coaxially shielded conductive yarns. Sci. Adv. 11, eadx4518. doi: 10.1126/sciadv.adx4518 41091868 PMC12525953

[B91] Lekadir K. Feragen A. Fofanah A. J. Frangi A. F. Buyx A. Emelie A. . (2024). FUTURE-AI: international consensus guideline for trustworthy and deployable artificial intelligence in healthcare. BMJ. 388, e081554. doi: 10.48550/arXiv.2309.12325 PMC11795397 39909534

[B92] Lewis P. Perez E. Piktus A. Petroni F. Karpukhin V. Goyal N. . (2020). Retrieval-augmented generation for knowledge-intensive nlp tasks. Adv. Neural Inf. Process. Syst. 33, 9459–9474.

[B93] Li X. Dunn J. Salins D. Zhou G. Zhou W. Schüssler-Fiorenza Rose S. M. . (2017). Digital health: tracking physiomes and activity using wearable biosensors reveals useful health-related information. PloS Biol. 15, e2001402. doi: 10.1371/journal.pbio.2001402 28081144 PMC5230763

[B94] Li Y. Lu F. Wang Q. Huang Q. Ling S. Xu N. . (2025). Digital intelligence for cardiometabolic health management in older adults: protocol for a community-based prospective cohort study. BMC Geriatr. 25, 826. doi: 10.1186/s12877-025-06548-7 41174586 PMC12577308

[B95] Liao P. Greenewald K. Klasnja P. Murphy S. (2020). Personalized HeartSteps: a reinforcement learning algorithm for optimizing physical activity. Proc. ACM Interact. Mob. Wearable Ubiquitous Technol. 4, 1–22. doi: 10.1145/3381007 34527853 PMC8439432

[B96] Lindholm M. E. Marabita F. Gomez-Cabrero D. Rundqvist H. Ekström T. J. Tegnér J. . (2014). An integrative analysis reveals coordinated reprogramming of the epigenome and the transcriptome in human skeletal muscle after training. Epigenetics 9, 1557–1569. doi: 10.4161/15592294.2014.982445 25484259 PMC4622000

[B97] Liu Y. Barker A. R. Li M. Gu Y. Williams C. A. (2025b). Validation of polar verity sense for heart rate monitoring during school-based high-intensity interval training in adolescents. J. Sports Sci. 43, 1076–1084. doi: 10.1080/02640414.2025.2491163 40227137

[B98] Liu X. Rivera S. C. Moher D. Calvert M. J. Denniston A. K. Ashrafian H. . (2020). Reporting guidelines for clinical trial reports for interventions involving artificial intelligence: the CONSORT-AI extension. Lancet Digit. Health 2, e537–e548. doi: 10.1016/S2589-7500(20)30218-1 33328048 PMC8183333

[B99] Liu X. Xu M. Wang H. Zhu L. (2025a). Integrating precision medicine and digital health in personalized weight management: the central role of nutrition. Nutrients 17, 2695. doi: 10.3390/nu17162695 40871725 PMC12389408

[B100] Liu X. Yang Y. Zong H. Zhang K. Jiang M. Yu C. . (2024). Core reference ontology for individualized exercise prescription. Sci. Data 11, 1349. doi: 10.1038/s41597-024-04217-9 39695140 PMC11655637

[B101] MaChado A. Ferreira D. F. Ferreira S. Almeida-Antunes N. Carvalho P. Melo P. . (2025). A systematic review of Chest-Worn sensors in cardiac assessment: Technologies, Advantages, and limitations. Sensors 25, 6049. doi: 10.3390/s25196049 41094872 PMC12526813

[B102] Mancia G. Brunström M. Burnier M. Grassi G. Januszewicz A. Muiesan M. L. . (2023). 2023 ESH guidelines for the management of arterial hypertension the Task Force for the management of arterial hypertension of the European Society of Hypertension endorsed by the European Renal Association (ERA) and the International Society of Hypertension (ISH). J. Hypertens. 41, 1874–2071. doi: 10.1097/HJH.0000000000003480 37345492

[B103] Martins M. F. Murry L. T. Telford L. Moriarty F. (2022). Direct-to-consumer genetic testing: an updated systematic review of healthcare professionals’ knowledge and views, and ethical and legal concerns. Eur. J. Hum. Genet. 30, 1331–1343. doi: 10.1038/s41431-022-01205-8 36220915 PMC9553629

[B104] Marzano-Felisatti J. M. De Lucca L. Priego-Quesada J. I. Pino-Ortega J. (2024). Heart rate measurement accuracy during intermittent efforts under laboratory conditions: a comparative analysis between chest straps and armband. Appl. Sci. 14, 11872. doi: 10.3390/app14241187230654563

[B105] Mason R. Pearson L. T. Barry G. Young F. Lennon O. Godfrey A. . (2023). Wearables for running gait analysis: a systematic review. Sports Med. 53, 241–268. doi: 10.1007/s40279-022-01760-6 36242762 PMC9807497

[B106] McMahan B. Moore E. Ramage D. Hampson S. y Arcas B. A. (2017). Communication-efficient learning of deep networks from decentralized data ( Pmlr). Available online at: https://proceedings.mlr.press/v54/mcmahan17a?ref=https://githubhelp.com (Accessed March 20, 2026).

[B107] Mejía-Mejía E. May J. M. Torres R. Kyriacou P. A. (2020). Pulse rate variability in cardiovascular health: a review on its applications and relationship with heart rate variability. Physiol. Meas. 41, 07TR01. doi: 10.1088/1361-6579/ab998c 32498055

[B108] Miller G. W. (2021). Integrating the exposome into a multi-omic research framework. Exposome 1, osab002. doi: 10.1093/exposome/osab002 37538530 PMC10399725

[B109] Mohr D. C. Schueller S. M. Montague E. Burns M. N. Rashidi P. (2014). The behavioral intervention technology model: an integrated conceptual and technological framework for eHealth and mHealth interventions. J. Med. Internet Res. 16, e146. doi: 10.2196/jmir.3077 24905070 PMC4071229

[B110] MoTrPAC Study Group, Lead Analysts, and MoTrPAC Study Group . (2024). Temporal dynamics of the multi-omic response to endurance exercise training. Nature 629, 174–183. doi: 10.1038/s41586-023-06877-w PMC11062907 38693412

[B111] Mühlen J. M. Stang J. Skovgaard E. L. Judice P. B. Molina-Garcia P. Johnston W. . (2021). Recommendations for determining the validity of consumer wearable heart rate devices: expert statement and checklist of the INTERLIVE network. Br. J. Sports Med. 55, 767–779. doi: 10.1136/bjsports-2020-103148 33397674 PMC8273688

[B112] Muñoz Fabra E. Díez J.-L. Bondia J. Laguna Sanz A. J. (2021). A comprehensive review of continuous glucose monitoring accuracy during exercise periods. Sens. 21, 479. doi: 10.3390/s21020479 33445438 PMC7828017

[B113] Muse E. D. Topol E. J. (2024). Transforming the cardiometabolic disease landscape: multimodal AI-powered approaches in prevention and management. Cell Metab. 36, 670–683. doi: 10.1016/j.cmet.2024.02.002 38428435 PMC10990799

[B114] Nahum-Shani I. Hekler E. B. Spruijt-Metz D. (2015). Building health behavior models to guide the development of just-in-time adaptive interventions: a pragmatic framework. Health Psychol. 34, 1209. doi: 10.1037/hea0000306 26651462 PMC4732268

[B115] Nahum-Shani I. Smith S. N. Spring B. J. Collins L. M. Witkiewitz K. Tewari A. . (2018). Just-in-time adaptive interventions (JITAIs) in mobile health: key components and design principles for ongoing health behavior support. Ann. Behav. Med. 52, 446–462. doi: 10.1007/s12160-016-9830-8 27663578 PMC5364076

[B116] Nasb M. Dayoub L. Chen N. (2025). Precision prevention in exercise: integrating OMICS biomarkers and artificial intelligence for injury risk assessment. Advanced Exercise Health Sci. 2, 241–253. doi: 10.1016/j.aehs.2025.09.00142574925

[B117] NHGRI (2023). DNA Sequencing Costs: Data. Available online at: https://www.genome.gov/about-genomics/fact-sheets/DNA-Sequencing-Costs-Data (Accessed January 29, 2026).

[B118] Omar M. Dinalankara W. Mulder L. Coady T. Zanettini C. Imada E. L. . (2023). Using biological constraints to improve prediction in precision oncology. Available online at: https://www.cell.com/iscience/fulltext/S2589-0042(23)00185-2?uuid=uuid%3Aaf9fdf4e-08f9-4844-841b-d08a0e142645 (Accessed July 29, 2026). 10.1016/j.isci.2023.106108 PMC9958363 36852282

[B119] Onorati F. Regalia G. Caborni C. LaFrance W. C. Blum A. S. Bidwell J. . (2021). Prospective study of a multimodal convulsive seizure detection wearable system on pediatric and adult patients in the epilepsy monitoring unit. Front. Neurol. 12, 724904. doi: 10.3389/fneur.2021.724904 34489858 PMC8418082

[B120] Ooka T. (2025). The era of preemptive medicine: developing medical digital twins through omics, IoT, and AI integration. JMA J. 8, 1–10. doi: 10.31662/jmaj.2024-0213 PMC11799569 39926086

[B121] Organization (2020). WHO Guidelines on Physical Activity and Sedentary Behaviour ( World Health Organization). Available online at: https://books.google.com/books?hl=zh-CN&lr=&id=QI5FEAAAQBAJ&oi=fnd&pg=PR4&dq=+the+WHO+Guidelines+on+Physical+Activity+and+Sedentary+Behaviour&ots=9-ZSoO57H4&sig=C_NUuQZ2RQXFoQ66wzGhgHMYSt8 (Accessed July 27, 2026).

[B122] Organization (2022a). Global Status Report on Physical Activity 2022: Country Profiles ( World Health Organization). Available online at: https://books.google.com/books?hl=zh-CN&lr=&id=zxmsEAAAQBAJ&oi=fnd&pg=PA2&dq=Global+Status+Report+on+Physical+Activity+2022.+Geneva:+World+Health+Organization.+ISBN:+978-9&ots=GqmZ5pdl8p&sig=yKVQKhME-VT2iM6rMCTneDMws28 (Accessed July 28, 2026).

[B123] Organization (2022b). Invisible Numbers: The True Extent of Noncommunicable Diseases and What to do About Them ( World Health Organization). Available online at: https://books.google.com/books?hl=zh-CN&lr=&id=iHsOEQAAQBAJ&oi=fnd&pg=PR5&dq=Invisible+Numbers:+The+True+Extent+of+Noncommunicable+Diseases+and+What+to+Do+About+Them.+Geneva:+World+Health+Organization.+ISBN:+978-92-4-005766-1&ots=B4ybgGJlWg&sig=KqeWl6zHKs7diIYa7v1U0Lv5XL4 (Accessed July 28, 2026).

[B124] Organization (2023). Regulatory Considerations on Artificial Intelligence for Health ( World Health Organization). Available online at: https://books.google.com/books?hl=zh-CN&lr=&id=YHoOEQAAQBAJ&oi=fnd&pg=PR5&dq=World+Health+Organization.+(2023).+Regulatory+Considerations+on+Artificial+Intelligence+for+Health.+Geneva:+World+Health+Organization.+ISBN:+978-92-4-007887-1.&ots=WplpxTxqTS&sig=zOk_XtTMD76kls43TL60j_tdUp8 (Accessed July 28, 2026).

[B125] Organization (2025). Global Strategy on Digital Health 2020-2027 ( World Health Organization). Available online at: https://books.google.com/books?hl=zh-CN&lr=&id=_DWcEQAAQBAJ&oi=fnd&pg=PA2&dq=Global+Strategy+on+Digital+Health+2020%E2%80%932027.+Geneva:+World+Health+Organization.+ISBN:+978-92-4-011687-0&ots=rVtHcys6JY&sig=EBF-V0unMjx2xjLYNqld_OelpIg (Accessed July 28, 2026).

[B126] Ozemek C. Bonikowske A. Christle J. Gallo P. (2025). ACSM’s Guidelines for Exercise Testing and Prescription ( Lippincott Williams & Wilkins). Available online at: https://books.google.com/books?hl=zh-CN&lr=&id=lIk_EQAAQBAJ&oi=fnd&pg=PT12&dq=ACSM%E2%80%99s+Guidelines+for+Exercise+Testing+and+Prescription.+12th+ed.+Ozemek,+C.+Bonikowske,+A.R.+Christle,+J.W.+and+Gallo,+P.M.+(eds.).+Philadelphia,+PA:+Wolters+Kluwer.&ots=3EmQohMo3m&sig=hd2mqk_B3T6JH2KFSgoj0cI5kYs (Accessed July 27, 2026).

[B127] Pannunzio V. Kleinsmann M. Snelders D. Raijmakers J. (2023). From digital health to learning health systems: four approaches to using data for digital health design. Health Syst. 12, 481–494. doi: 10.1080/20476965.2023.2284712 38235300 PMC10791080

[B128] Pasadyn S. R. Soudan M. Gillinov M. Houghtaling P. Phelan D. Gillinov N. . (2019). Accuracy of commercially available heart rate monitors in athletes: a prospective study. Cardiovasc. diagnosis Ther. 9, 379. doi: 10.21037/cdt.2019.06.05 31555543 PMC6732081

[B129] Patten T. Preble E. A. Master H. Adjemian J. Ramirez A. McClain J. . (2026). The All of Us Research Program’s wearables dataset. Nat. Med. 32, 2302–2310. doi: 10.1038/s41591-026-04352-3 42045581 PMC13278962

[B130] Payne R. A. Symeonides C. N. Webb D. J. Maxwell S. R. J. (2006). Pulse transit time measured from the ECG: an unreliable marker of beat-to-beat blood pressure. J. Appl. Physiol. 100, 136–141. doi: 10.1152/japplphysiol.00657.2005 16141378

[B131] Pearl J. (2010). Causal inference. Causality: object. Assess. 6, 39–58. doi: 10.7551/mitpress/11252.003.004442438465

[B132] Perez M. V. Mahaffey K. W. Hedlin H. Rumsfeld J. S. Garcia A. Ferris T. . (2019). Large-scale assessment of a smartwatch to identify atrial fibrillation. N. Engl. J. Med. 381, 1909–1917. doi: 10.1056/NEJMoa1901183 31722151 PMC8112605

[B133] Petek B. J. Al-Alusi M. A. Moulson N. Grant A. J. Besson C. Guseh J. S. . (2023). Consumer wearable health and fitness technology in cardiovascular medicine. J. Am. Coll. Cardiol. 82, 245–264. doi: 10.1016/j.jacc.2023.04.054 37438010 PMC10662962

[B134] Piccialli F. Chiaro D. Sarwar S. Cerciello D. Qi P. Mele V. (2025). AgentAI: a comprehensive survey on autonomous agents in distributed AI for industry 4.0. Expert Syst. Appl. 291, 128404. doi: 10.1016/j.eswa.2025.12840442574925

[B135] Pichon M. Bouleti C. Hery-Arnaud G. Burucoa C. (2025). Heterogeneity and lack of standardisation in gut microbiome testing: a comparative assessment of French medical biology laboratories. Gut. 75(7), 1454–1456. doi: 10.1136/gutjnl-2025-336981 41115749 PMC13311931

[B136] Porcari S. Mullish B. H. Asnicar F. Ng S. C. Zhao L. Hansen R. . (2025). International consensus statement on microbiome testing in clinical practice. Lancet Gastroenterol. Hepatol. 10, 154–167. doi: 10.1016/S2468-1253(24)00311-X 39647502 PMC12343204

[B137] Prince S. A. Adamo K. B. Hamel M. Hardt J. Connor Gorber S. Tremblay M. (2008). A comparison of direct versus self-report measures for assessing physical activity in adults: a systematic review. Int. J. Behav. Nutr. Phys. Act. 5, 56. doi: 10.1186/1479-5868-5-56 18990237 PMC2588639

[B138] Qian T. Yoo H. Klasnja P. Almirall D. Murphy S. A. (2021). Estimating time-varying causal excursion effects in mobile health with binary outcomes. Biometrika 108, 507–527. doi: 10.1093/biomet/asaa070 34629476 PMC8494142

[B139] Regalia G. Onorati F. Lai M. Caborni C. Picard R. W. (2019). Multimodal wrist-worn devices for seizure detection and advancing research: focus on the Empatica wristbands. Epilepsy Res. 153, 79–82. doi: 10.1016/j.eplepsyres.2019.02.007 30846346

[B140] Rivera S. C. Liu X. Chan A.-W. Denniston A. K. Calvert M. J. Ashrafian H. . (2020). Guidelines for clinical trial protocols for interventions involving artificial intelligence: the SPIRIT-AI extension. Lancet Digit. Health 2, e549–e560. doi: 10.1136/bmj.m3210 33328049 PMC8212701

[B141] Rojas-Macias M. A. Mariethoz J. Andersson P. Jin C. Venkatakrishnan V. Aoki N. P. . (2019). Towards a standardized bioinformatics infrastructure for N- and O-glycomics. Nat. Commun. 10, 3275. doi: 10.1038/s41467-019-11131-x 31332201 PMC6796180

[B142] Ross R. Goodpaster B. H. Koch L. G. Sarzynski M. A. Kohrt W. M. Johannsen N. M. . (2019). Precision exercise medicine: understanding exercise response variability. Br. J. Sports Med. 53, 1141–1153. doi: 10.1136/bjsports-2018-100328 30862704 PMC6818669

[B143] Rotzler S. Krshiwoblozki M. V. Schneider-Ramelow M. (2021). Washability of e-textiles: current testing practices and the need for standardization. Text. Res. J. 91, 2401–2417. doi: 10.1177/0040517521996727

[B144] Sanford J. A. Nogiec C. D. Lindholm M. E. Adkins J. N. Amar D. Dasari S. . (2020). Molecular transducers of physical activity consortium (MoTrPAC): mapping the dynamic responses to exercise. Cell. 181, 1464–1474. doi: 10.1016/j.cell.2020.06.004 32589957 PMC8800485

[B145] Santiago-Rodriguez T. M. Hollister E. B. (2021). Multi ‘omic d/ata integration: A review of concepts, considerations, and approaches. Seminars in Perinatology 45(6), 151456. doi: 10.1016/j.semperi.2021.151456 34256961

[B146] Santos V. M. Gomes B. B. Neto M. A. Amaro A. M. (2024). A systematic review of insole sensor technology: recent studies and future directions. Appl. Sci. 14, 6085. doi: 10.3390/app1414608530654563

[B147] Schelling X. Robertson S. (2020). A development framework for decision support systems in high-performance sport. Int. J. Comput. Sci. Sport 19, 1–23. doi: 10.2478/ijcss-2020-0001

[B148] Schumann M. Doherty C. (2024). Bridging gaps in wearable technology for exercise and health professionals: a brief review. Int. J. Sports Med. 45, 949–957. doi: 10.1055/a-2376-6332 39079705

[B149] Schweizer T. Gilgen-Ammann R. (2025). Wrist-worn and arm-worn wearables for monitoring heart rate during sedentary and light-to-vigorous physical activities: device validation study. JMIR cardio 9, e67110. doi: 10.2196/67110 40116771 PMC11951816

[B150] Schyvens A.-M. Peters B. Van Oost N. C. Aerts J.-M. Masci F. Neven A. . (2025). A performance validation of six commercial wrist-worn wearable sleep-tracking devices for sleep stage scoring compared to polysomnography. SLEEP Adv. 6, zpaf021. doi: 10.1093/sleepadvances/zpaf021 40303381 PMC12038347

[B151] Seshadri D. R. Li R. T. Voos J. E. Rowbottom J. R. Alfes C. M. Zorman C. A. . (2019). Wearable sensors for monitoring the internal and external workload of the athlete. NPJ Digital Med. 2, 71. doi: 10.1038/s41746-019-0149-2 31372506 PMC6662809

[B152] Shaban-Nejad A. Michalowski M. Bianco S. (2023). Multimodal AI in Healthcare: A Paradigm Shift in Health Intelligence. Eds. Shaban-Nejad A. Michalowski M. Bianco S. (Cham: Springer International Publishing). doi: 10.1007/978-3-031-14771-5

[B153] Shaffer F. Ginsberg J. P. (2017). An overview of heart rate variability metrics and norms. Front. Public Health 5, 290215. doi: 10.3389/fpubh.2017.00258 29034226 PMC5624990

[B154] Shen M. Chen S. Ding X. (2024). The effectiveness of digital twins in promoting precision health across the entire population: a systematic review. NPJ Digital Med. 7, 145. doi: 10.1038/s41746-024-01146-0 38831093 PMC11148028

[B155] Shiffman S. Stone A. A. Hufford M. R. (2008). Ecological momentary assessment. Annu. Rev. Clin. Psychol. 4, 1–32. doi: 10.1146/annurev.clinpsy.3.022806.091415 18509902

[B156] Singh B. Ahmed M. Staiano A. E. Gough C. Petersen J. Vandelanotte C. . (2024). A systematic umbrella review and meta-meta-analysis of eHealth and mHealth interventions for improving lifestyle behaviours. NPJ Digital Med. 7, 179. doi: 10.1038/s41746-024-01172-y 38969775 PMC11226451

[B157] Singstad B.-J. Azulay N. Bjurstedt A. Bjørndal S. S. Drageseth M. F. Engeset P. . (2021). Estimation of heart rate variability from finger photoplethysmography during rest, mild exercise and mild mental stress. J. Electr. Bioimpedance 12, 89–102. doi: 10.2478/joeb-2021-0012 35069945 PMC8713388

[B158] Steinhubl S. R. Waalen J. Edwards A. M. Ariniello L. M. Mehta R. R. Ebner G. S. . (2018). Effect of a home-based wearable continuous ECG monitoring patch on detection of undiagnosed atrial fibrillation: the mSToPS randomized clinical trial. JAMA 320, 146–155. doi: 10.1001/jama.2018.8102 29998336 PMC6583518

[B159] Su C. Wang P. Foo N. Ho D. (2025). Optimizing metabolic health with digital twins. NPJ Aging 11, 20. doi: 10.1038/s41514-025-00211-6 40128254 PMC11933362

[B160] Subramanian I. Verma S. Kumar S. Jere A. Anamika K. (2020). Multi-omics data integration, interpretation, and its application. Bioinf. Biol. Insights 14, 117793221989905. doi: 10.1177/1177932219899051 32076369 PMC7003173

[B161] Sutton R. T. Pincock D. Baumgart D. C. Sadowski D. C. Fedorak R. N. Kroeker K. I. (2020). An overview of clinical decision support systems: benefits, risks, and strategies for success. NPJ Digital Med. 3, 17. doi: 10.1038/s41746-020-0221-y 32047862 PMC7005290

[B162] Svensson T. Madhawa K. Chung U. Svensson A. K. (2024). Validity and reliability of the Oura Ring Generation 3 (Gen3) with Oura sleep staging algorithm 2.0 (OSSA 2.0) when compared to multi-night ambulatory polysomnography: A validation study of 96 participants and 421,045 epochs. Sleep Med. 115, 251–263. doi: 10.1016/j.sleep.2024.01.020 38382312

[B163] Takeuchi H. Ishizawa T. Kishi A. Nakamura T. Yoshiuchi K. Yamamoto Y. (2024). Just-in-time adaptive intervention for stabilizing sleep hours of Japanese workers: microrandomized trial. J. Med. Internet Res. 26, e49669. doi: 10.2196/49669 38861313 PMC11200036

[B164] Takeuchi H. Suwa K. Kishi A. Nakamura T. Yoshiuchi K. Yamamoto Y. (2022). The effects of objective push-type sleep feedback on habitual sleep behavior and momentary symptoms in daily life: mHealth intervention trial using a health care internet of things system. JMIR mHealth uHealth 10, e39150. doi: 10.2196/39150 36201383 PMC9585447

[B165] Tao L. Li H. (2025). Real-time health monitoring for athletes in dynamic sports environments based on split federated learning and recurrent neural networks. IEEE Trans. Consum. Electron. 71(3), 8956–8967. doi: 10.1109/TCE.2025.358720025079929

[B166] Tewari A. Murphy S. A. (2017). “ From ads to interventions: contextual bandits in mobile health,” in Mobile Health. Eds. Rehg J. M. Murphy S. A. Kumar S. ( Springer International Publishing, Cham), 495–517. doi: 10.1007/978-3-319-51394-2_25

[B167] Tian W. Lin R. Zheng H. Yang Y. Wu G. Zhang Z. . (2025). SportsGPT: an LLM-driven framework for interpretable sports motion assessment and training guidance. doi: 10.48550/arXiv.2512.14121

[B168] Torres-Ronda L. Beanland E. Whitehead S. Sweeting A. Clubb J. (2022). Tracking systems in team sports: A narrative review of applications of the data and sport specific analysis. Sports Med. - Open 8, 15. doi: 10.1186/s40798-022-00408-z 35076796 PMC8789973

[B169] Vaid A. Lampert J. Lee J. Sawant A. Apakama D. Sakhuja A. . (2024). Natural language programming in medicine: administering evidence based clinical workflows with autonomous agents powered by generative large language models. doi: 10.48550/arXiv.2401.02851

[B170] Vallée A. (2024). Envisioning the future of personalized medicine: role and realities of digital twins. J. Med. Internet Res. 26, e50204. doi: 10.2196/50204 38739913 PMC11130780

[B171] Van Eetvelde H. Mendonça L. D. Ley C. Seil R. Tischer T. (2021). Machine learning methods in sport injury prediction and prevention: a systematic review. J. Exp. Orthop. 8, 27. doi: 10.1186/s40634-021-00346-x 33855647 PMC8046881

[B172] Vasey B. Nagendran M. Campbell B. Clifton D. A. Collins G. S. Denaxas S. . (2022). Reporting guideline for the early stage clinical evaluation of decision support systems driven by artificial intelligence: DECIDE-AI. bmj 377, e070904. doi: 10.1136/bmj-2022-070904 35584845 PMC9116198

[B173] Vorisek C. N. Lehne M. Klopfenstein S. A. I. Mayer P. J. Bartschke A. Haese T. . (2022). Fast healthcare interoperability resources (FHIR) for interoperability in health research: systematic review. JMIR Med. Inf. 10, e35724. doi: 10.2196/35724 35852842 PMC9346559

[B174] Vvedenskaya O. Holčapek M. Vogeser M. Ekroos K. Meikle P. J. Bendt A. K. (2022). Clinical lipidomics – A community-driven roadmap to translate research into clinical applications. J. Mass Spectrom. Adv. Clin. Lab. 24, 1–4. doi: 10.1016/j.jmsacl.2022.02.002 35199094 PMC8844780

[B175] Wang L. Ma C. Feng X. Zhang Z. Yang H. Zhang J. . (2024). A survey on large language model based autonomous agents. Front. Comput. Sci. 18, 186345. doi: 10.1007/s11704-024-40231-130311153

[B176] Wenderott K. Krups J. Zaruchas F. Weigl M. (2024). Effects of artificial intelligence implementation on efficiency in medical imaging—a systematic literature review and meta-analysis. NPJ Digital Med. 7, 265. doi: 10.1038/s41746-024-01248-9 39349815 PMC11442995

[B177] WHO (2025). Global Strategy on Digital Health 2020-2027 ( World Health Organization). Available online at: https://books.google.com/books?hl=zh-CN&lr=&id=_DWcEQAAQBAJ&oi=fnd&pg=PA2&dq=Global+strategy++on+digital+health++2020-2027&ots=rVsCaEz_L_&sig=wvM_nGLPtETwi8YD8sa9hwONSbk (Accessed February 3, 2026).

[B178] Wilkinson M. D. Dumontier M. Aalbersberg I. J. Appleton G. Axton M. Baak A. . (2016). The FAIR Guiding Principles for scientific data management and stewardship: Comment. Sci. Data 3, 160018. doi: 10.1038/sdata.2016.18 26978244 PMC4792175

[B179] Wilson S. Tolley C. Mc Ardle R. Lawson L. Beswick E. Hassan N. . (2024). Recommendations to advance digital health equity: a systematic review of qualitative studies. NPJ Digital Med. 7, 173. doi: 10.1038/s41746-024-01177-7 38951666 PMC11217442

[B180] Woolley S. I. Collins T. Mitchell J. Fredericks D. (2019). Investigation of wearable health tracker version updates. BMJ Health Care Inform. 26, e100083. doi: 10.1136/bmjhci-2019-100083 31597642 PMC7062347

[B181] Wu X. Liu Y.-K. Iliuk A. B. Tao W. A. (2023). Mass spectrometry-based phosphoproteomics in clinical applications. TrAC Trends Anal. Chem. 163, 117066. doi: 10.1016/j.trac.2023.117066 37215489 PMC10195102

[B182] Yazdani A. Boerwinkle E. (2014). Causal inference in the age of decision medicine. J. Data Min. Genom. proteom. 6, 163. doi: 10.4172/2153-0602.1000163 26085955 PMC4466903

[B183] Yu H. Kotlyar M. Dufresne S. Thuras P. Pakhomov S. (2022). “ Feasibility of using an armband optical heart rate sensor in naturalistic environment”, in: Biocomputing 2023: Proceedings of the Pacific Symposium (Kohala Coast, Hawaii, USA: WORLD SCIENTIFIC), 43–54. doi: 10.1142/9789811270611_0005 PMC9830591 36540963

[B184] Yuda E. Yamamoto K. Yoshida Y. Hayano J. (2020). Differences in pulse rate variability with measurement site. J. Physiol. Anthropol. 39, 4. doi: 10.1186/s40101-020-0214-1 32085811 PMC7035641

[B185] Zhang S. Wang Z. Wang Y. Zhu Y. Zhou Q. Jian X. . (2024b). A metabolomic profile of biological aging in 250,341 individuals from the UK Biobank. Nat. Commun. 15, 8081. doi: 10.1038/s41467-024-52310-9 39278973 PMC11402978

[B186] Zhang G. Xu Z. Jin Q. Chen F. Fang Y. Liu Y. . (2025). Leveraging long context in retrieval augmented language models for medical question answering. NPJ Digital Med. 8, 239. doi: 10.1038/s41746-025-01651-w 40316710 PMC12048518

[B187] Zhang K. Zhou H.-Y. Baptista-Hon D. T. Gao Y. Liu X. Oermann E. . (2024a). Concepts and applications of digital twins in healthcare and medicine. Patterns 5, 101028. doi: 10.1016/j.patter.2024.101028 39233690 PMC11368703

[B188] Ziegler D. M. Stiennon N. Wu J. Brown T. B. Radford A. Amodei D. . (2020). Fine-tuning language models from human preferences. doi: 10.48550/arXiv.1909.08593

