# Supplementary material for: WMRE2030: integrating wearable devices, multi-omics, and artificial intelligence–driven real-time feedback into a daily-scale closed-loop framework for a new era of precision exercise
Source: Front Physiol. 2026 Sep 21;17:1883295. doi: 10.3389/fphys.2026.1883295 (PMC13635893; doi:10.3389/fphys.2026.1883295)
Supplement: Supplementary file 1 [file Table1.docx]

Supplementary Material

# 1 Supplementary Tables

## 1.1 Supplementary Table 1

**Supplementary Tables 1.** Annual publication counts of WMRS2030-related domains retrieved from OpenAlex and PubMed (2010–2025)

| Year | AI (Web of Science) | Multi-omics (Web of Science) | Wearables (Web of Science) | Precision exercise (Web of Science) |
| --- | --- | --- | --- | --- |
| Web of Science |  |  |  |  |
| 2010 | 6648 | 72696 | 94 | 94 |
| 2011 | 7588 | 78738 | 109 | 112 |
| 2012 | 8135 | 86515 | 121 | 113 |
| 2013 | 9170 | 89626 | 168 | 134 |
| 2014 | 10234 | 95082 | 270 | 169 |
| 2015 | 11460 | 99682 | 505 | 174 |
| 2016 | 13541 | 106183 | 909 | 183 |
| 2017 | 17724 | 109455 | 1340 | 198 |
| 2018 | 27968 | 115858 | 1893 | 245 |
| 2019 | 46257 | 132770 | 2552 | 264 |
| 2020 | 70578 | 144364 | 3226 | 345 |
| 2021 | 100486 | 162148 | 4122 | 386 |
| 2022 | 128082 | 164889 | 4578 | 449 |
| 2023 | 141254 | 156055 | 4818 | 501 |
| 2024 | 172909 | 167740 | 5725 | 643 |
| 2025 | 224891 | 185602 | 7209 | 1028 |
| Scopus |  |  |  |  |
| 2010 | 44817 | 155187 | 364 | 157 |
| 2011 | 46219 | 153660 | 531 | 221 |
| 2012 | 48902 | 163057 | 528 | 237 |
| 2013 | 48531 | 170623 | 678 | 237 |
| 2014 | 49535 | 213141 | 1466 | 275 |
| 2015 | 54016 | 244498 | 3281 | 287 |
| 2016 | 63576 | 241225 | 4144 | 314 |
| 2017 | 82143 | 244162 | 5537 | 322 |
| 2018 | 109490 | 248858 | 6087 | 432 |
| 2019 | 155582 | 268140 | 7119 | 454 |
| 2020 | 195731 | 288962 | 7874 | 504 |
| 2021 | 245758 | 316763 | 9019 | 652 |
| 2022 | 296851 | 312811 | 10567 | 751 |
| 2023 | 362162 | 296588 | 11929 | 762 |
| 2024 | 461367 | 327199 | 13789 | 907 |
| 2025 | 546713 | 360231 | 16758 | 1436 |
| PubMed |  |  |  |  |
| 2010 | 1955 | 384 | 70 | 87 |
| 2011 | 2301 | 416 | 93 | 129 |
| 2012 | 2483 | 489 | 93 | 128 |
| 2013 | 2944 | 579 | 107 | 139 |
| 2014 | 3457 | 672 | 196 | 172 |
| 2015 | 4153 | 765 | 402 | 205 |
| 2016 | 5084 | 980 | 692 | 219 |
| 2017 | 6965 | 1129 | 914 | 240 |
| 2018 | 11260 | 1377 | 1239 | 282 |
| 2019 | 17532 | 1808 | 1606 | 297 |
| 2020 | 26915 | 2507 | 2089 | 362 |
| 2021 | 40026 | 3721 | 2629 | 477 |
| 2022 | 52654 | 4871 | 3034 | 534 |
| 2023 | 60132 | 5681 | 3154 | 571 |
| 2024 | 73378 | 7583 | 3655 | 682 |
| 2025 | 101302 | 13639 | 4878 | 1119 |
| OpenAlex |  |  |  |  |
| 2010 | 54376 | 218894 | 878 | 750 |
| 2011 | 60957 | 243570 | 1100 | 905 |
| 2012 | 67457 | 274186 | 1281 | 1064 |
| 2013 | 75949 | 300014 | 1670 | 1174 |
| 2014 | 83852 | 322566 | 3000 | 1377 |
| 2015 | 93069 | 340047 | 5041 | 1538 |
| 2016 | 109622 | 359746 | 6852 | 1802 |
| 2017 | 141131 | 388832 | 8491 | 1912 |
| 2018 | 203635 | 424867 | 11019 | 2222 |
| 2019 | 280487 | 479557 | 13592 | 2663 |
| 2020 | 371304 | 573498 | 17185 | 3294 |
| 2021 | 465195 | 610988 | 20883 | 3784 |
| 2022 | 543237 | 632830 | 24810 | 4247 |
| 2023 | 722265 | 737559 | 30745 | 5887 |
| 2024 | 736200 | 499731 | 24002 | 5038 |
| 2025 | 913012 | 485859 | 24489 | 5631 |

## 2.1 Supplementary Table 2

**Supplementary Table 2.** Predefined scoring criteria and evidence supporting the relative technological maturity and cost accessibility of major omics layers

**Panel A. Rating methodology and operational definitions**

| **Dimension** | **Rating** | **Operational definition** |
| --- | --- | --- |
| Technological maturity | 1/5 (★☆☆☆☆) | Predominantly exploratory; limited standardization, quality-control consensus, reproducibility evidence, or access outside specialist laboratories. |
| Technological maturity | 2/5 (★★☆☆☆) | Specialized research application with established proof of concept, but substantial technical, reproducibility, interoperability, or deployment constraints. |
| Technological maturity | 3/5 (★★★☆☆) | Established research use with recognizable workflows and service availability, but limited routine translational deployment or persistent interpretative/standardization limitations. |
| Technological maturity | 4/5 (★★★★☆) | Mature and broadly available research implementation, including large-cohort or multicentre use, with remaining context-dependent, cross-platform, or interpretative limitations. |
| Technological maturity | 5/5 (★★★★★) | Highly standardized, population-scale, widely deployable technology with mature quality-control pipelines and broad institutional or commercial availability. |
| Cost accessibility | 1/5 (★☆☆☆☆) | Very limited accessibility because of very high direct cost, invasive or complex sampling, specialized infrastructure, low throughput, and/or scarce service availability. |
| Cost accessibility | 2/5 (★★☆☆☆) | Low accessibility; relatively high per-sample and infrastructure costs, substantial preprocessing or analytical burden, and limited scalability. |
| Cost accessibility | 3/5 (★★★☆☆) | Moderate accessibility; feasible in established research facilities, but cost, sample handling, infrastructure, or downstream analysis remains a meaningful barrier. |
| Cost accessibility | 4/5 (★★★★☆) | High accessibility; moderate pricing, scalable workflows, and broad core-facility availability, although depth, sample type, or analytical scope can materially increase total cost. |
| Cost accessibility | 5/5 (★★★★★) | Very high relative accessibility; low-to-moderate representative price, low-burden sampling or one-time acquisition, strong batch scalability, and broad service availability. |

**Panel B. Layer-specific ratings, implementation rationale, and supporting evidence**

| **Omics layer** | **Technological maturity** | **Maturity criteria and layer-specific rationale** | **Supporting evidence** | **Cost accessibility** | **Representative cost and accessibility rationale** | **Cost source and access date** |
| --- | --- | --- | --- | --- | --- | --- |
| **Genomics** | **5/5 (★★★★★)** | • Sequencing and SNP-genotyping platforms are highly standardized, with mature quality-control and variant-calling workflows.  • Widely implemented in clinical genomics, biobanks, population cohorts, and commercial testing services.  • Stable genomic information is generally acquired once, supporting long-term stratification and model calibration. | • [NHGRI DNA Sequencing Costs](https://www.genome.gov/sequencingcosts)  *Accessed 29 July 2026.* | **5/5 (★★★★★)** | • Representative prices: whole-genome sequencing approximately US$300–1,000 per genome; SNP arrays approximately US$40–100 per sample.  • Strong batch scalability and broad institutional/commercial availability.  • One-time baseline sampling reduces repeated longitudinal burden; cost varies substantially by sequencing depth and analysis package. | • [NHGRI DNA Sequencing Cost Data](https://www.genome.gov/sequencingcosts)  *Accessed 29 July 2026.* |
| **Epigenomics** | **4/5 (★★★★☆)** | • DNA-methylation arrays, particularly Infinium MethylationEPIC platforms, are widely used in epigenome-wide association studies.  • Laboratory and bioinformatic workflows are mature, but tissue specificity, batch effects, platform transitions, and longitudinal interpretation remain important constraints.  • Best positioned for periodic calibration and contextual biological characterization rather than routine high-frequency decision-making. | • [Illumina Infinium MethylationEPIC platform](https://www.illumina.com/products/by-type/microarray-kits/infinium-methylation-epic.html)  *Accessed 29 July 2026.* | **3/5 (★★★☆☆)** | • Representative methylation-array price: approximately US$300–450 per sample, depending on batch size and service configuration.  • Requires DNA processing, batch planning, specialist quality control, and downstream bioinformatic analysis.  • Accessible through major core facilities, but less routine and less price-accessible than genotyping or 16S microbiome profiling. | • [SciLifeLab NGI Illumina array price list](https://ngisweden.scilifelab.se/wp-content/uploads/2022/09/PriceList_NGI_IlluminaArrays_ValidFrom_Aug1st_2022-1.pdf)  *Accessed 29 July 2026.* |
| **Transcriptomics** | **4/5 (★★★★☆)** | • RNA sequencing is an established standard for transcriptome profiling, with mature library-preparation, sequencing, and analytical pipelines.  • Broadly available through institutional and commercial facilities and used extensively in mechanistic and translational research.  • Pre-analytical sensitivity, tissue dependence, temporal variability, and bioinformatic complexity limit routine individual-level interpretation. | • [RNA-seq methodological reference (PubMed)](https://pubmed.ncbi.nlm.nih.gov/26813401)  *Accessed 29 July 2026.* | **4/5 (★★★★☆)** | • Representative RNA-sequencing price: approximately US$200–400 per sample, depending on sequencing depth and library design.  • High-throughput batch processing is widely available.  • Total cost may increase because of stringent RNA handling, library preparation, storage, and downstream analysis requirements. | • [MDC Berlin sequencing service pricing](https://www.mdc-berlin.de/media/39941)  *Accessed 29 July 2026.* |
| **Proteomics** | **3/5 (★★★☆☆)** | • Mass-spectrometry and affinity-based platforms are well established and increasingly used in biomarker discovery and large cohorts.  • Coverage, normalization, batch correction, cross-platform comparability, and validation of candidate markers remain methodologically demanding.  • Current use remains more strongly anchored to research and validation settings than routine precision-exercise services. | • [Olink Explore platform overview](https://olink.com/products/olink-explore-series)  *Accessed 29 July 2026.* | **2/5 (★★☆☆☆)** | • Representative multiplex-proteomics price: approximately US$500–1,500 per sample, depending on panel breadth and platform.  • Requires specialized instrumentation or proprietary panels, rigorous sample processing, and substantial analytical support.  • Costs rise rapidly with proteome coverage, validation requirements, and repeated measurements. | • [Vanderbilt High-Throughput Biomarker Core pricing](https://medsites.vumc.org/highthroughputbiomarkercore/pricing)  *Accessed 29 July 2026.* |
| **Metabolomics** | **3/5 (★★★☆☆)** | • Mass-spectrometry and NMR metabolomics are widely used in systems biology, epidemiology, and population cohorts, including UK Biobank.  • Targeted assays may be highly reproducible, whereas untargeted workflows remain affected by pre-analytical variation, incomplete annotation, and platform dependence.  • The field is moving toward broader deployment, but routine individual-level interpretation remains less standardized than genomics or transcriptomics. | • [UK Biobank metabolomics category](https://biobank.ndph.ox.ac.uk/ukb/label.cgi?id=220)  *Accessed 29 July 2026.* | **4/5 (★★★★☆)** | • Representative price: approximately US$100–850 per sample, depending on targeted versus untargeted design and analytical breadth.  • Targeted panels can be relatively affordable and batch-scalable through established core facilities.  • Untargeted profiling, compound annotation, and downstream interpretation can substantially increase the total cost. | • [EMBL Metabolomics Core service fees](https://www.embl.org/groups/metabolomics/service-fees-external-users/)  *Accessed 29 July 2026.* |
| **Microbiome** | **4/5 (★★★★☆)** | • 16S rRNA sequencing and shotgun metagenomics are widely used in international research initiatives and large cohort studies.  • Sampling is non-invasive and sequencing workflows are mature, with broad service availability.  • Differences in collection, storage, DNA extraction, sequencing pipelines, batch effects, and causal interpretation remain important cross-study limitations. | • [NIH Human Microbiome Project](https://commonfund.nih.gov/hmp)  *Accessed 29 July 2026.* | **5/5 (★★★★★)** | • Representative prices: 16S rRNA sequencing approximately US$55–75 per sample; shotgun metagenomics approximately US$200–250 per sample.  • Stool sampling is non-invasive, self-collection is feasible, and commercial/core-facility services are widely available.  • Bioinformatic processing and interpretation add cost, but the direct assay remains relatively accessible and scalable. | • [MD Anderson Microbiome Core services and fees](https://www.mdanderson.org/research/research-resources/core-facilities/microbiome-core-facility/services-and-fees.html)  *Accessed 29 July 2026.* |

Each omics layer was evaluated across five technological-maturity domains (platform/protocol standardization, quality-control and analytical workflow maturity, reproducibility and cross-platform comparability, scale of research or translational use, and availability beyond specialized laboratories) and five cost-accessibility domains (representative direct per-sample price, sampling and preprocessing burden, infrastructure intensity, batch scalability, and availability through commercial or institutional core facilities). The overall rating was assigned from the rounded median of the domain-level assessments. When evidence was heterogeneous, two adjacent categories were equally plausible, or a critical implementation bottleneck remained, the lower category was retained as a conservative classification. The ratings are evidence-informed comparative categories and are not a formally validated health-technology-assessment index. Prices are approximate publicly available service prices in US dollars and should not be interpreted as universal market averages. Actual costs vary by assay depth, platform, batch size, geographical region, institutional subsidy, shipping, sample preparation, and whether bioinformatic analysis is included. Star ratings summarize relative real-world implementation readiness across the six omics layers considered in Figure 3; they do not represent clinical utility, biological importance, or a validated quantitative index. Where evidence was heterogeneous, the more conservative category was retained. NMR, nuclear magnetic resonance; RNA-seq, RNA sequencing; SNP, single-nucleotide polymorphism; US$, United States dollars; WGS, whole-genome sequencing.

## 2.2 Supplementary Table 3

**Supplementary Table 3.** Standardized Prompt-Engineering Protocol for a Minimum-Viable WMRE2030 Large Language Model Demonstrator

| **Module** | **Standardized prompt instruction (copy verbatim)** | **Operational purpose** |
| --- | --- | --- |
| **1. System role and scope** | You are a WMRE2030-guided precision-exercise decision-support demonstrator. Organize information according to the WMRE2030 day-scale closed-loop framework: state sensing, constraint interpretation, decision support, exercise execution, and response feedback. Your role is to support understanding and structured reasoning, not to diagnose disease, replace a qualified professional, or generate autonomous clinical exercise prescriptions. Treat every output as exploratory and provisional. | Defines the model identity, intended use, and non-clinical boundary. |
| **2. Reader goal and context** | First identify the reader’s primary goal, population category, training status, available resources, time constraints, equipment, preferences, acceptable cost, and level of professional supervision. Ask only for information that is necessary to address the stated goal. Do not assume that a missing variable is normal or favourable. | Anchors the interaction in the reader’s own goals and real-world constraints. |
| **3. Framework constraint layer** | Use the WMRE2030 article as the principal methodological constraint. Maintain the sequence: (1) state sensing; (2) interpretation of biological, behavioural, environmental, and resource constraints; (3) evidence-grounded decision support; (4) exercise execution; and (5) response feedback. Apply the principle “same architecture, different parameters”: retain the same functional loop across general, clinical, and athletic populations, while adapting objectives, thresholds, data requirements, automation, and professional oversight. | Prevents the model from reducing WMRE2030 to generic fitness advice. |
| **4. Temporal and information architecture** | Classify available information into: (a) relatively stable background information, such as age, medical and injury history, long-term training history, baseline fitness, and genomic information when legitimately available; (b) periodically updated information, such as laboratory biomarkers, body composition, performance tests, and dynamic omics; (c) high-frequency or daily information, such as physical activity, sleep, heart rate, heart-rate variability, training load, perceived exertion, fatigue, soreness, stress, readiness, and environmental constraints; and (d) exercise-execution and response information, including prescribed and completed exercise, adherence, actual intensity and volume, symptoms, and subsequent recovery. Treat these streams as asynchronous and complementary rather than requiring identical sampling frequencies. | Operationalizes the multi-timescale WMRE2030 data structure. |
| **5. Dynamic evidence layer** | When verified literature-retrieval tools are available, retrieve evidence that matches the reader’s population, goal, intervention, outcome, and risk profile. Prioritize: current population-specific guidelines; recognized professional consensus or position statements; systematic reviews and meta-analyses; high-quality randomized or controlled trials; and other evidence only when stronger evidence is unavailable. Appraise population match, intervention match, recency, directness, consistency, and uncertainty. Cite only sources that can be verified. Never invent a citation, DOI, sample size, effect estimate, or recommendation. When retrieval is unavailable, state this explicitly and do not imply that current evidence has been searched. | Creates an evidence-grounded layer while limiting hallucinated references. |
| **6. Gate 1—data integrity** | Before updating any recommendation, examine signal loss, motion artefact, physiologically implausible values, timestamp misalignment, device malfunction, insufficient wear time, unexplained missingness, and changes in device, firmware, or processing algorithm. If data quality is inadequate, suspend model-based updating, identify the affected variables, and either maintain the previous acceptable plan or use a conservative fallback. Do not fabricate missing measurements. | Prevents low-quality sensor inputs from driving new recommendations. |
| **7. Gate 2—model and evidence applicability** | Determine whether the reader, data, and requested task fall within the populations, measurement ranges, and contexts supported by the available evidence. Identify conflicting inputs, out-of-scope requests, important missing variables, and high uncertainty. When applicability is uncertain, reduce automation, avoid progression, maintain the current plan or revert to a conservative evidence-based option, and explain the source of uncertainty. | Checks distributional and contextual fit before decision support. |
| **8. Gate 3—physiological risk** | Screen for concerning symptoms, abnormal physiological values, acute illness or injury indicators, unusually poor recovery, excessive fatigue or soreness, persistent adverse trends, and repeated safety-threshold violations. Safety must take precedence over performance optimization. When risk signals are present, recommend reducing, postponing, or suspending exercise as appropriate, shorten the validity period of any advice, avoid diagnosis, and advise suitable professional evaluation. | Places safety ahead of optimization and defines conservative fallback behaviour. |
| **9. Gate 4—human escalation** | Initiate human review when abnormalities persist, multiple risk signals cluster, clinical red flags are reported, uncertainty cannot be resolved, the individual belongs to a higher-risk clinical population, or an incorrect recommendation could cause meaningful harm. Direct the reader to the most relevant qualified professional, such as a physician, clinical exercise professional, physiotherapist, sport scientist, or qualified coach. Do not continue autonomous progression after an escalation trigger. | Defines explicit conditions for professional oversight. |
| **10. Day-scale decision process** | After applying the four safety gates, summarize the current state by separating observed facts, longitudinal trends, evidence-based interpretation, and uncertain inference. Then select one decision action: Progress; Maintain; Reduce or modify; or Suspend and escalate. The day scale is the organizing and reassessment horizon; it does not require all data to be collected daily or the plan to be changed every day. | Converts the framework into a transparent, repeatable decision cycle. |
| **11. Supportive exercise plan** | When the decision is within scope and risk is acceptable, provide a cautious and executable support plan using relevant FITT-VP elements: frequency, intensity, time, type, volume, progression, and recovery. State the monitoring variables, stopping conditions, and period of validity. Avoid false numerical precision. Clearly distinguish evidence-supported recommendations from model inference and from the reader’s own observed response. | Produces practical support without overstating precision or authority. |
| **12. Feedback and iterative updating** | Specify the next feedback cycle: what should be recorded, when reassessment should occur, which responses would support progression, which would support maintenance or regression, and which would trigger professional review. Incorporate completed exercise, actual load, adherence, subjective response, objective response, and adverse events into the next cycle. Do not interpret non-adherence as physiological non-response. | Closes the loop and prevents conflation of implementation failure with biological response. |
| **13. Required output format** | Return the response under the following headings: (1) Reader goal and context; (2) Data quality and completeness; (3) Current-state summary; (4) Four safety-gate assessment; (5) Evidence summary and applicability; (6) Decision—Progress, Maintain, Reduce or modify, or Suspend and escalate; (7) Supportive exercise plan; (8) Safety boundaries and stopping conditions; (9) Uncertainty and limitations; and (10) Next feedback cycle. Keep the reasoning concise, traceable, and understandable to the intended reader. | Standardizes outputs for comparison, auditing, and reader comprehension. |
| **14. Mandatory closing statement** | End every response with: “This output is an exploratory, WMRE2030-guided decision-support demonstration. It does not constitute autonomous medical diagnosis, clinical exercise prescription, or a substitute for qualified professional judgement.” | Maintains a visible and consistent use limitation. |

**Use:** Upload the WMRE2030 article, then copy the prompt modules below into the model in numerical order. The protocol is intended for exploratory reader interaction and methodological demonstration only; it is not a trained, externally validated, or clinically evaluated exercise-prescription system. *FITT-VP* frequency, intensity, time, type, volume, and progression; *LLM* large language model; *WMRE2030* Wearables, Multi-Omics, AI-Driven Real-Time Feedback, and Exercise 2030. This protocol is intended to structure reader interaction with a general-purpose or locally deployed LLM; it does not modify model parameters and should not be described as model fine-tuning unless parameter-efficient or full-parameter training is actually performed.

## 2.3 Supplementary Table 4

**Supplementary Table 4.** Standardized Reader Input Template for the Minimum-Viable WMRE2030 Demonstrator

| **Input domain** | **Information to provide** | **Entry instruction** |
| --- | --- | --- |
| Goal and intended use | Primary health, fitness, rehabilitation, or performance goal; desired time horizon; expected type of support. | Describe one primary goal first. Separate general health support from performance optimization or clinically supervised exercise. |
| Personal background | Age; sex; height; body mass; occupation or daily activity context; relevant preferences. | Provide only information necessary for the stated goal. |
| Health and risk context | Known conditions; medications; current symptoms; previous injuries; pregnancy status where relevant; professional clearance; contraindications. | Do not upload information that you do not wish to process. Report current warning symptoms before requesting exercise progression. |
| Training background | Current frequency; training history; recent detraining; primary exercise modes; technical proficiency; recent competition or major event. | Include dates or approximate time periods where possible. |
| Baseline assessments | Cardiorespiratory fitness; resting heart rate; blood pressure; strength, power, mobility, body-composition, laboratory, or biological assessments. | State the assessment date, device or method, units, and whether testing was professionally supervised. |
| Wearable and digital data | Device brand and model; wear location; firmware or software version; valid wear days; resting heart rate; HRV; sleep; activity; training load; other available metrics. | Report missing data, changes in device or algorithm, unusual readings, and known measurement limitations. |
| Current daily state | Sleep quality and duration; fatigue; soreness; pain; stress; motivation; perceived recovery; illness symptoms; environmental and scheduling constraints. | Use consistent scales across days and define the scale anchors. |
| Recent exercise execution | Planned session; completed session; exercise type; duration; intensity; volume; adherence; session RPE; symptoms during exercise; recovery after exercise. | Separate the prescribed plan from what was actually completed. |
| Resource and access constraints | Available time; equipment; financial constraints; internet or platform access; professional supervision; travel; climate; cultural or personal preferences. | State which constraints are fixed and which are negotiable. |
| Feedback for the next cycle | Actual load; adherence; subjective response; objective response; adverse events; reasons for modification or non-completion. | Provide feedback after the exercise period specified in the previous output; do not omit adverse or unexpected responses. |

*HRV* heart-rate variability; *RPE* rating of perceived exertion; *WMRE2030* Wearables, Multi-Omics, AI-Driven Real-Time Feedback, and Exercise 2030. Readers should avoid entering directly identifying or unnecessary sensitive information into third-party systems and should follow applicable privacy and data-governance requirements.
